# Causal factors for migraine in Mendelian randomization studies: a systematic review and meta-analysis

**DOI:** 10.3389/fneur.2025.1660995

**Published:** 2025-09-09

**Authors:** Xinyao Li, Qingming Liu, Huitong Ni, Jiaqi Ni, Shu Yang, Jianguang Ji

**Affiliations:** ^1^West China School of Pharmacy, Sichuan University, Chengdu, China; ^2^Department of Pharmacy/Evidence-Based Pharmacy Center, West China Second University Hospital, Sichuan University, Chengdu, China; ^3^Key Laboratory of Drug-Targeting and Drug Delivery System of the Education Ministry and Sichuan Province, West China School of Pharmacy, Sichuan University, Chengdu, China; ^4^Sichuan Engineering Laboratory for Plant-Sourced Drug and Sichuan Research Center for Drug Precision Industrial Technology, West China School of Pharmacy, Sichuan University, Chengdu, China; ^5^Department of Public Health and Medicinal Administration, University of Macau, Macau SAR, China

**Keywords:** migraine, Mendelian randomization, causal relationship, systematic review, meta-analysis

## Abstract

**Background:**

Migraine is a familial, episodic disorder characterized by complex sensory processing dysfunction, with headache serving as its hallmark feature. While numerous risk factors have been proposed, the causal nature of these associations often remains ambiguous. Mendelian randomization (MR) represents a robust epidemiological framework that leverages genetic variants to infer causal relationships, thereby overcoming limitations of observational studies. This study systematically reviews and meta-analyzes MR evidence to elucidate bidirectional causal relationships between migraine and systemic diseases, identify novel risk determinants, and highlight critical gaps for future mechanistic investigations.

**Methods:**

A comprehensive literature search was conducted across seven databases (PubMed, Embase, Cochrane Library, Web of Science, China National Knowledge Infrastructure, WanFang Data Knowledge Service Platform, and VIP China Science and Technology Journal Database) using predefined search strategies and exclusion criteria. The search time limit was from the construction of the database to July 3, 2024. Study eligibility was independently assessed by two reviewers, with data extraction processes adhering to STROBE-MR guidelines. Included studies were evaluated for quality using validated criteria, and relevant data (study design, participant demographics, genetic instruments, analytical methods, and outcomes) were systematically extracted. Data synthesis involved meta-analytical pooling of effect estimates using Review Manager 5.4, with forest plots generated to visualize results. Causal relationships were interpreted according to the WHO ICD-11 disease classification system, with subgroup analyses performed for migraine with aura (MWA) and migraine without aura (MOA).

**Results:**

A total of 60 studies involving 331 MR analyses were included, revealing bidirectional causal relationships between migraine and multiple phenotypes: migraine was identified as a causal factor for 6 diseases (Alzheimer’s disease, cervical artery dissection, venous thromboembolism, coronary artery disease, angina, large artery stroke), 3 behavioral habits (delayed age at first sexual intercourse, maternal smoking, reduced physical activity), 1 dietary intakes (alcohol consumption), and 3 physiological indicators (elevated interleukin-2, increased Body Mass Index, higher serum vitamin D levels) (*p* < 0.05). Conversely, 6 diseases (venous thromboembolism, breast cancer, insomnia, difficulty awakening, major depressive disorder, depression), 5 behavioral factors (television watching, smoking initiation, delayed AFS, more schooling, reduced physical activity), 4 dietary determinants (coffee, alcohol, cheese, salad intake), 13 physiological parameters (hemostatic, cardiovascular, metabolic, and genetic markers), and 1 gut microbiota taxon (*LachnospiraceaeUCG001*) were causal determinants of migraine risk (*p* < 0.05). Subtype-specific analyses showed MOA was causally associated with 4 diseases (AD, CeAD, CAD, LAS) and delayed AFS as an exposure, and influenced by breast cancer, celiac disease, TV watching, delayed AFS, increased schooling, and physiological parameters (DBP, PP, serum calcium, IGF-1) as an outcome; MWA demonstrated causal relationships with CeAD and LAS as an exposure, and associations with VTE, SLE, MDD, delayed AFS, coffee intake, and hemostatic markers as an outcome (*p* < 0.05 for all).

**Conclusion:**

This systematic review provides robust genetic evidence supporting bidirectional causal relationships between migraine and multiple phenotypes, including systemic diseases, behavioral habits, dietary factors, and physiological parameters. Subtype-specific analyses highlight distinct causal pathways for MOA and MWA, underscoring the clinical heterogeneity of migraine. These findings advance our understanding of migraine pathogenesis and inform precision medicine approaches, while also identifying novel therapeutic targets for this disabling condition. More data will be needed in the future to obtain a more specific assessment.

**Systematic review registration:**

https://www.crd.york.ac.uk/PROSPERO/view/CRD42025636141, Identifier CRD42025636141.

## 1 Introduction

Migraine represents the most prevalent neurological disorder in primary care settings, accounting for over 90% of headache-related consultations according to epidemiological studies (1, 2). As highlighted by the Global Burden of Disease study, it ranks as the second leading cause of disability globally and the primary cause among young women (3). With a prevalence of 18% in females and 6% in males, migraine imposes substantial societal and economic burdens, particularly chronic migraine, which affects 2% of the global population (4).

Clinically defined by severe throbbing, unilateral headache accompanied by nausea, photophobia, and vomiting, migraine pathogenesis involves trigeminovascular system activation modulated by genetic and environmental factors (5). The disorder is classified into two primary forms: migraine with aura (MWA) and migraine without aura (MOA), with further categorization into chronic or episodic subtypes. Hemiplegic migraine, a rare variant, manifests as transient unilateral weakness and sensory disturbances (6). Recent genome-wide association studies have identified 28 genetic loci associated with headache phenotypes, including 14 previously linked to migraine. Notably, these studies uncovered significant brain-specific genetic correlations (7).

The primary objective of migraine management is to mitigate attack severity and duration (8). Pharmacological interventions encompass a diverse range of agents, including acetaminophen, triptans, nonsteroidal anti-inflammatory drugs (NSAIDs) such as ibuprofen and diclofenac potassium, dihydroergotamine, non-opioid analgesics, NSAID-triptan combinations, and antiemetics (9).

Mendelian randomization (MR) leverages genetic variants as instrumental variables (IVs) to infer causal relationships between exposure and outcome traits (83, 84). By capitalizing on genetic epidemiology, MR circumvents limitations inherent in observational studies through the random allocation of genotypes during meiosis (10). Valid IVs must satisfy three key assumptions: (1) robust association with the exposure of interest, (2) independence from confounding variables, and (3) exclusive mediation of outcomes via the exposure. A repertoire of MR methods, including inverse variance weighting (IVW), MR-Egger, weighted median, and mode-based approaches, are employed to validate causal inferences (11).

This review systematically synthesizes evidence from MR studies on migraine to explore bidirectional causal relationships between migraine and other diseases. The objectives are twofold: (1) to characterize the causal associations between migraine and its subtypes with various phenotypes, and (2) to advance therapeutic target identification for migraine management. By integrating genetic evidence, this review aims to clarify migraine’s role as both a risk factor and potential outcome of systemic disorders, thereby informing mechanistic research and novel drug development.

## 2 Materials and methods

The study group conducted the systematic review following the criteria of the Preferred Reporting Items for Systematic Reviews and Meta-Analyses (PRISMA) statement (12), and it was registered in the International Prospective Register of Systematic Reviews (PROSPERO), CRD42025636141, 9 January 2025.

### 2.1 Search strategy

To identify MR studies on migraine, the study group performed a systematic literature search in PubMed, Embase, Cochrane Library, Web of Science, China National Knowledge Infrastructure (CNKI), WanFang Data Knowledge Service Platform (WanFang), and VIP China Science and Technology Journal Database (VIP). A combination of subject terms and free words was used for the search. The English search terms included Mendelian randomization, migraine, hemicrania, cephalalgia, headache, cephalodynia, cranial pain, and head pain, and the Chinese search terms included Mendelian Randomization and migraine. The system searched the literature in the above databases that used Mendelian randomization to study the correlation between migraine and disease phenotypes. The search time limit was from the construction of the database to July 3, 2024.

### 2.2 Criteria for inclusion and exclusion of literature

#### 2.2.1 Inclusion criteria

(1) All published papers using MR to explore the causal relationship between migraine and multiple exposures or diseases, including unidirectional or bidirectional MR studies.
(2) Associations reported as Odds Ratios (OR) and 95%confidence intervals (95% CIs) using instrumental variable methods.

#### 2.2.2 Exclusion criteria

(1) Articles unrelated to the topic were excluded.
(2) Any case reports, narrative reviews, letters, editorials, opinions, incomplete manuscripts, and conference abstracts were excluded.
(3) Articles in languages other than Chinese or English were excluded.
(4) Articles without extractable indicators were excluded.
(5) When there are multiple publications based on the same Genome-Wide Association Studies (GWAS) (same participants), only publications with the largest sample size and the latest published study (if the sample size is the same) are included, without sample size limitations.

### 2.3 Study selection and data extraction

Relevant article data were systematically retrieved from databases, downloaded, and imported into the reference management software Endnote X9. Duplicate references were automatically removed using the software. Two reviewers (XL and QL) independently screened the title and abstract of all retrieved articles after employing the search strategy. Studies included after screening were retrieved for full-text review. Disagreements were resolved by consulting a third reviewer (JN). The original author was contacted for incomplete literature if needed.

A data extraction form was created to extract the following information from each study: (1) general information about the article: title, author, publication year, abstract; (2) basic information about the research: research methods, exposure and outcome, study population, sample size, exposure GWAS data source, outcome GWAS data source, main causal effect estimation method; (3) outcomes: number of single nucleotide polymorphisms (SNPs), computed OR values, 95% CI, *p*-values; and (4) other information required by the Strengthening the Reporting of Observational Studies in Epidemiology using Mendelian Randomization (STROBE-MR) guidelines, for example, sensitivity and additional analysis. Microsoft Excel 2019 was used to extract data. Two reviewers (XL and QL) independently extracted data from the studies included. Disagreements were resolved by consulting a third reviewer (SY). IVW is the traditional MR method for converting the composite ratio estimate for each SNP into an overall estimate, and it is also the method used to select the extraction results when extracting the data in this study.

### 2.4 Evaluation of literature quality

To evaluate the quality of the Mendelian randomization (MR) studies incorporated in the systematic review, the STROBE-MR guidelines (13, 14) were employed as a tool for literature quality assessment. This quality assessment guideline takes into consideration both the research methodology and reporting standards, enabling a fair evaluation of potentially biased studies. The guideline is structured into several sections: title and abstract (item 1), introduction (items 2–3), methods (items 4–9), results (items 10–13), discussion (items 14–17), and other information (items 18–20). Items 1 to 14 within these entries were adapted from the assessment entries in the study by Ibrahim et al. (15). These entries encompassed title and abstract, background, objectives, study design and data sources, statistical methods for main analyses, software and preregistration, descriptive data, main results, sensitivity analyses and additional analyses, key results, limitations, interpretation, generalizability, and the core assumptions of Mendelian randomization. Ibrahim et al. adjusted and utilized these quality assessment scores in MR meta-analyses and systematic reviews. They converted the scores into percentages, with scores below 80% indicating a high risk of bias, scores between 80 and 90% representing a medium risk of bias, and scores above 90% signifying a low risk of bias. Two reviewers (XL and HN) independently evaluated the risk of bias for the included studies and cross-checked the results. Any disagreements were resolved through discussion with a third reviewer (SY). The initial quality assessment scheme is provided in Supplementary Table 2.

### 2.5 Classification of research themes

The themes of studies included in the literature were categorized according to the International Classification of Diseases, 11th Revision (ICD-11) for Mortality and Morbidity Statistics issued by the World Health Organization (WHO) (16). Each disease was searched in ICD-11, and the largest submenu containing the disease was selected to represent the system where the disease is located. Categorize them into: mental, behavioral or neurodevelopmental disorders; diseases of the circulatory system; sleep–wake disorders; diseases of the digestive system; diseases of the skin; endocrine, nutritional or metabolic diseases; diseases of the nervous system; neoplasms; diseases of the immune system; diseases of the respiratory system; diseases of the ear or mastoid process; diseases of the genitourinary system; and other ICD-11 diseases, 13 categories in total. After categorizing the included literature according to the ICD-11 classification, there were still some non-disease studies that were difficult to organize, so the authors of this paper categorized them as “non-disease” and subdivided the “non-disease” factors into behavioral habits non-disease factors, dietary intake non-disease factors, physiologic non-disease factors, and other non-disease factors. When encountering the same indicator with different names in different literature, we combined them into one name (e.g., coffee intake and coffee consumption were merged into coffee consumption).

### 2.6 Statistical analysis

When two or more MR estimates could be obtained based on the same results from non-overlapping samples, a meta-analysis was performed by Review Manage 5.4 software to obtain a combined estimate, generate forest plots, and the choice of model was decided based on the value of I2. We used OR and 95% CI as our effect indicators. Heterogeneity was assessed using I2, with I2 values between 25 and 50% considered mild heterogeneity, I2 values between 50 and 75% moderate heterogeneity, and I2 values greater than 75% considered severe heterogeneity. A random-effects model was selected when I2 was greater than 50%, and a fixed-effects model was selected when it was less than or equal to 50%. *p* < 0.05 was considered a statistically significant difference.

## 3 Results

### 3.1 Results of the literature search

Based on the established literature search strategy, the search was conducted in the corresponding databases according to the search formula, and the search results are shown in Supplementary Table 1.

**Figure 1:**
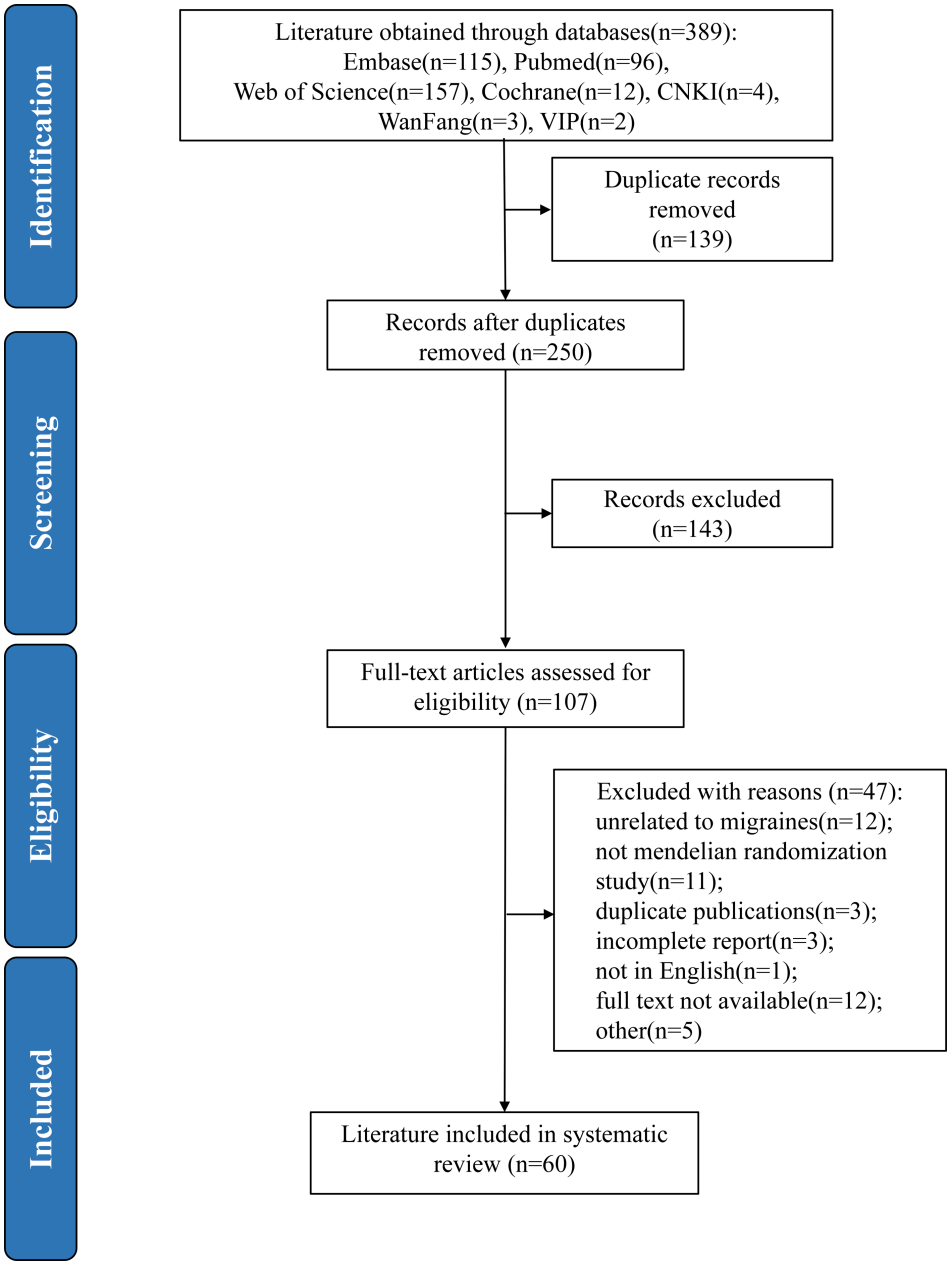
PRISMA flow diagram of literature search. * CNKI, China National Knowledge Infrastructure; WanFang, WanFang Data Knowledge Service Platform; VIP, VIP China Science and Technology Journal Database; PRISMA, Preferred Reporting Items for Systematic Reviews and Meta-Analyses.

A total of 389 literature articles were retrieved through the above search formula to study migraine using MR methods, and after removing duplicate records through Endnote X9, 250 articles were left, and 60 articles were finally obtained by reading the titles and abstracts for initial screening, and then by reading the full text to screen out the unavailability of the full text/missing the screening of the literature, 60 literature were finally included. The literature screening process is shown in Figure 1.

### 3.2 General information about the studies included

A total of 60 original journal articles were included, with publication years from the year of the library’s creation to July 3, 2024. The general information of the included literature is shown in Table 1.

**Table 1:** General information about the studies included.

Study (first author, year)	Method	Theme	Ethnicity of exposure	Ethnicity of outcome	Whether causality exists		
					Forward	Reverse	Subtypes
Peter Yin, 2017 (56)	Two-sample unidirectional MR	Elevation of serum calcium levels by 1 mg/dL	European	European	No causal effect	Risk factor	Elevation of serum calcium levels by 1 mg/dL-MOA: risk factor
Johnsen, M. B, 2018 (57)	One-sample unidirectional MR	Smoking	Norway	Norway	No causal effect	/	/
Daghlas, I, 2020 (1) (28)	Two-sample unidirectional MR	AD, intelligence, brain volume	European	European	No causal effect	/	/
Daghlas, I, 2020 (2) (23)	Two-sample bidirectional MR	Insomnia, difficulty awakening	European	European	No causal effect	Risk factors	/
Daghlas, I, 2020 (3) (17)	Two-sample unidirectional MR	CAD, myocardial infarction, angina, AF	European	European	Protective factors: CAD, myocardial infarction, angina No causal effect: AF	/	MOA-CAD: protective factor MWA-CAD: no causal effect
Emmanuel O. Adewuyi, 2020 (38)	Two-sample bidirectional MR	Endometriosis	European (approximately 93%) and Japanese ancestries (from Australia, Belgium, Denmark, Iceland, Japan, the UK, and the USA)	European	No causal effect	No causal effect	/
Guo, Y, 2020 (58)	Two-sample bidirectional MR	BP	European	European	No causal effect	Risk factors: SBP, DBP, PP	DBP-MOA: risk factors PP-MOA: risk factors
Chu, S, 2021 (24)	Two-sample bidirectional MR	Insomnia	European	European	No causal effect	Risk factor	/
Guo, Y, 2021 (59)	Two-sample bidirectional MR	Hemostatic profile	European	European	No causal effect	Risk factors: FVIII activity, vWF levels, phosphorylated fibrinopeptide A Protective factors: fibrinogen levels, APTT	Fibrinogen levels-MWA: protective factor FVIII activity-MWA: risk factor vWF levels-MWA: risk factor APTT-MWA: protective factor Phosphorylated fibrinopeptide A-MWA: risk factor Fibrinogen levels-MOA: no causal effect
Brittany L Mitchell, 2022 (41)	Two-sample bidirectional MR	ICV	European	European	No causal effect	Protective factor	/
Chen, H, 2022 (60)	Two-sample unidirectional MR	Coffee consumption	British	European	No causal effect	/	No causal effect
Daghals, I, 2022 (21)	Two-sample unidirectional MR	CeAD, LAS	European	European	Risk factor: CeAD Protective factor: LAS	/	MOA-CeAD: risk factor MOA-LAS: protective factor MWA-CeAD: risk factor MWA-LAS: protective factor
Islam, M. R, 2022 (27)	Two-sample bidirectional MR	T2D	European	European	No causal effect	No causal effect	/
Keon-Joo Lee, 2022 (18)	Two-sample unidirectional MR	Stroke, ischemic stroke, hemorrhagic stroke	European	European	No causal effect	/	No causal effect
Mei-Jun Shu, 2022 (19)	Two-sample unidirectional MR	Ischemic stroke	European	European	No causal effect	/	/
Peng-Peng Niu, 2022 (42)	Two-sample bidirectional MR	Higher serum vitamin D levels	European	European	Risk factor	Protective factor	No causal effect
Reziya Abuduxukuer, 2022 (43)	Two-sample bidirectional MR	IGF-1	European (94.3%)	European	No causal effect	Protective factor	IGF-1-MWA: no causal effect IGF1-MOA: protective factor
Shuai Yuan, 2022 (61)	Two-sample bidirectional MR	Alcohol consumption, coffee consumption, smoking initiation, smoking index	European	European	Protective factor: alcohol consumption	Risk factors: smoking initiation Protective factors: alcohol consumption, coffee consumption No causal effect: smoking index	/
Bi, Y, 2023 (71)	Two-sample unidirectional MR	Genetic instrumental variables for lipids and lipid modifying targets	N/A	European (92.55%)	/	Protective factor: APO-A1	/
Chong Fu, 2023 (44)	Two-sample bidirectional MR	Inflammatory cytokines	Finnish descent	European	Migraine-IL-2: protective factor	HGF-migraine: risk factor	/
Fang, T, 2023 (35)	Two-sample unidirectional MR	Breast cancer	European	European	/	Risk factor	Breast cancer-MWA: no causal effect Breast cancer-MOA: risk factor
Guo, X, 2023 (62)	Two-sample bidirectional MR	Total cortical SA, average cortical thickness, GMV, WMH, HV	European	European	No causal effect	Protective factors: SA, HV	/
Huo, J, 2023 (45)	Two-sample bidirectional MR	WMLs	European	European	No causal effect	No causal effect	/
Horton, M. K, 2023 (29)	Two-sample unidirectional MR	MS	European	N/A	No causal effect	/	/
He, Q, 2023 (46)	Two-sample bidirectional MR	Gut microbiota	European	European	N/A	N/A	N/A
Hua Xue, 2023 (30)	Two-sample unidirectional MR	AD	European	European	No causal effect	/	/
Hui Zheng,2023 (63)	Two-sample unidirectional MR	More years of schooling	European	European	/	Protective factors	More years of schooling-MOA: protective factor
Jin, C, 2023 (81)	Two-sample unidirectional MR	Tea intake	European	European	/	No causal effect	No causal effect
Lei Zhao, 2023 (47)	Two-sample bidirectional MR	WM	European	European	Established	Established	/
Mengmeng Wang, 2023 (20)	Two-sample unidirectional MR	Ischemic stroke	European	European	No causal effect	/	No causal effect
Nike Zoe Welander, 2023 (25)	Two-sample bidirectional MR	IBD, celiac disease	European	European	No causal effect	No causal effect	Celiac disease-MOA: protective factor
Tao Wei, 2023 (64)	Two-sample unidirectional MR	Neuralized E3 ubiquitin-protein ligase 1	European	European	/	Protective factor	/
Wenqiang Zhang, 2023 (39)	Two-sample bidirectional MR	CKD	European	European and Japanese ancestries	No causal effect	No causal effect	/
Xinhui Liu, 2023 (48)	Two-sample bidirectional MR	83 dietary habits	European	European	Include only supported hypotheses	Include only supported hypotheses	/
Xiaofeng Lv, 2023 (51)	Two-sample bidirectional MR	MDD	European	European	No causal effect	Risk factor	MDD-MWA: risk factor
Zhen-Ni Zhao, 2023 (40)	Two-sample bidirectional MR	PD	European	European	No causal effect	No causal effect	/
Baranova, A, 2024 (31)	Two-sample unidirectional MR	AD	European	European	Risk factor	/	/
Chengfeng Xu, 2024 (32)	Two-sample unidirectional MR	AD	European	European	Risk factor	/	/
Chengcheng Zhang, 2024 (72)	Two-sample unidirectional MR	Blood cis-eQTL, brain cis-eQTL	European	European	/	/	
Danfeng Xu, 2024 (54)	Two-sample unidirectional MR	SLE	European	European	/	No causal effect	SLE-MWA: risk factor SLE-MOA: no causal effect
Geng, C, 2024 (33)	Two-sample bidirectional MR	AD	European	European	Risk factor	No causal effect	/
Guanglu Li, 2024 (26)	Two-sample bidirectional MR	Psoriasis, T1D, RA, SLE, AR, asthma	European	European	No causal effect	No causal effect	No causal effect
Guoliang Zhu, 2024 (65)	Two-sample bidirectional MR	Delayed AFS	European	European	Protective factor	Protective factor	MOA-AFS: protective factor AFS-MWA: protective factor AFS-MOA: protective factor
Hao Lv, 2024 (36)	Two-sample bidirectional MR	AR	European	European	No causal effect	No causal effect	No causal effect
Hong, P, 2024 (73)	Two-sample unidirectional MR	Lipid metabolism characteristics	European	European	/	/	/
Jianxiong Gui, 2024 (74)	Two-sample unidirectional MR	TWAS	N/A	European	/	Protective factor: *REV1* Risk factor: *SREBF2*	/
Jareebi, Mohammad A, 2024 (66)	Two-sample unidirectional MR	Smoking initiation, smoking intensity, maternal smoking, cheese intake, salad intake, coffee consumption, BMI, physical activity	European	European	/	Risk factor: maternal smoking Protective factors: coffee consumption, cheese intake, salad intake, BMI, physical activity	/
Jinjin Zhang, 2024 (67)	Two-sample unidirectional MR	Coffee intake	European	European	/	Protective factor	Coffee intake-MWA: protective factor Coffee intake-MOA: no causal effect
Kang Qu, 2024 (1) (49)	Two-sample bidirectional MR	Gut microbiota	European	European	No causal effect	Risk factor: *LachnospiraceaeUCG001*	/
Kang Qu, 2024 (2) (68)	Two-sample unidirectional MR	LDL-C, APOB, TC	European	European	/	No causal effect	/
Kangjia Zhang, 2024 (37)	Two-sample bidirectional MR	MD	European	European	No causal effect	No causal effect	/
Lei Zhao, 2024 (34)	Two-sample unidirectional MR	AD, VaD, FTD, LBD	European	European	Migraine-AD: risk factor	/	MOA-AD: risk factor
Meixuan Ren, 2024 (55)	Two-sample unidirectional MR	SLE	European	European	/	/	SLE-MWA: risk factor SLE-MOA: no causal effect
Peihong Li, 2024 (69)	Two-sample unidirectional MR	SBs	European	European	/	Risk factor: watching TV	Watching TV-MOA: risk factor
Peng-Peng Niu, 2024 (70)	Two-sample unidirectional MR	LRP11, ITIH1, ADGRF5	European	European	/	Protective factors: LRP11, ADGRF5 Risk factors: ITIH1	/
Xiangyue Meng, 2024 (50)	Two-sample unidirectional MR	Gut microbiota	European (78%)	European	No causal effect	Risk factor: *LachnospiraceaeUCG001*	/
Xu-Peng Wu, 2024 (53)	Two-sample bidirectional MR	VTE	European	European	Risk factor	Risk factors	VTE-MWA: risk factor
Ya Li, 2024 (82)	Two-sample unidirectional MR	Psoriasis	European	European	/	No causal effect	/
Yang Li, 2024 (52)	Two-sample unidirectional MR	Depression, MDD, insomnia, sleep duration, short sleep duration, daytime sleepiness, napping	European	European	/	Risk factors: depression, MDD, insomnia No causal effect: sleep duration, short sleep duration, daytime sleepiness, napping	/
Yang Wang, 2024 (22)	Two-sample bidirectional MR	VTE	European	European	Risk factors	Risk factors	/

* Directionality in this table: Forward: Indicates the causal effect of migraine on the specified disease. Reverse: Indicates the causal effect of the specified disease on migraine. ** N/A: Not Applicable. *** Established: A causal relationship is established. Since the data is complex, they will be shown later using descriptive language. AD, Alzheimer’s disease; CAD, coronary artery disease; AF, atrial fibrillation; BP, blood pressure; SBP, systolic blood pressure; DBP, diastolic blood pressure; PP, pulse pressure; FVIII, coagulation factor VIII; vWF, von Willebrand factor; APTT, activated partial thromboplastin time; ICV, intracranial volume; CeAD, cervical artery dissection; LAS, large artery stroke; T2D, type 2 Diabetes; IGF-1, insulin-like growth factor 1; APO-A1, Apolipoprotein A1; IL-2, Interleukin-2; HGF, hepatocyte growth factor; SA, surface area (cortical); GMV, gray matter volume; WMH, white matter hyperintensities; HV, Hippocampal volume; WMLs, white matter lesions; MS, Multiple sclerosis; WM, white matter; IBD, inflammatory bowel disease; CKD, chronic kidney disease; MDD, major depressive disorder; PD; periodontitis; eQTL, expression quantitative trait loci; SLE, systemic lupus erythematosus; T1D, type 1 diabetes; RA, rheumatoid arthritis; AR, allergic rhinitis; AFS, age at first sexual intercourse; TWAS, cross-tissue transcriptome association studies; BMI, body mass index; LDL-C, low-density lipoprotein cholesterol; APOB, apolipoprotein B; TC, total cholesterol; MD, Meniere’s disease; VaD, vascular dementia; FTD, frontotemporal dementia; LBD, Lewy body dementia; SBs, sedentary behaviors; LRP11, ITIH1, ADGRF5, proteins; VTE, Venous thromboembolism.

We also extracted the details of the included literature, including general information, exposure data, outcome data, and main analysis, the results are shown in Supplementary Table 4.

### 3.3 Results of the literature quality assessment

A total of 60 included literature were assessed by using the STROBE-MR checklist. Seventeen as low risk of bias, 31 as medium risk of bias, and 12 as high risk of bias after the quality assessment process. The specific assessment results of all the literature are shown in Supplementary Table 3.

### 3.4 Causal relationships between migraine and multiple diseases

#### 3.4.1 Diseases of the circulatory system

**Figure 2:**
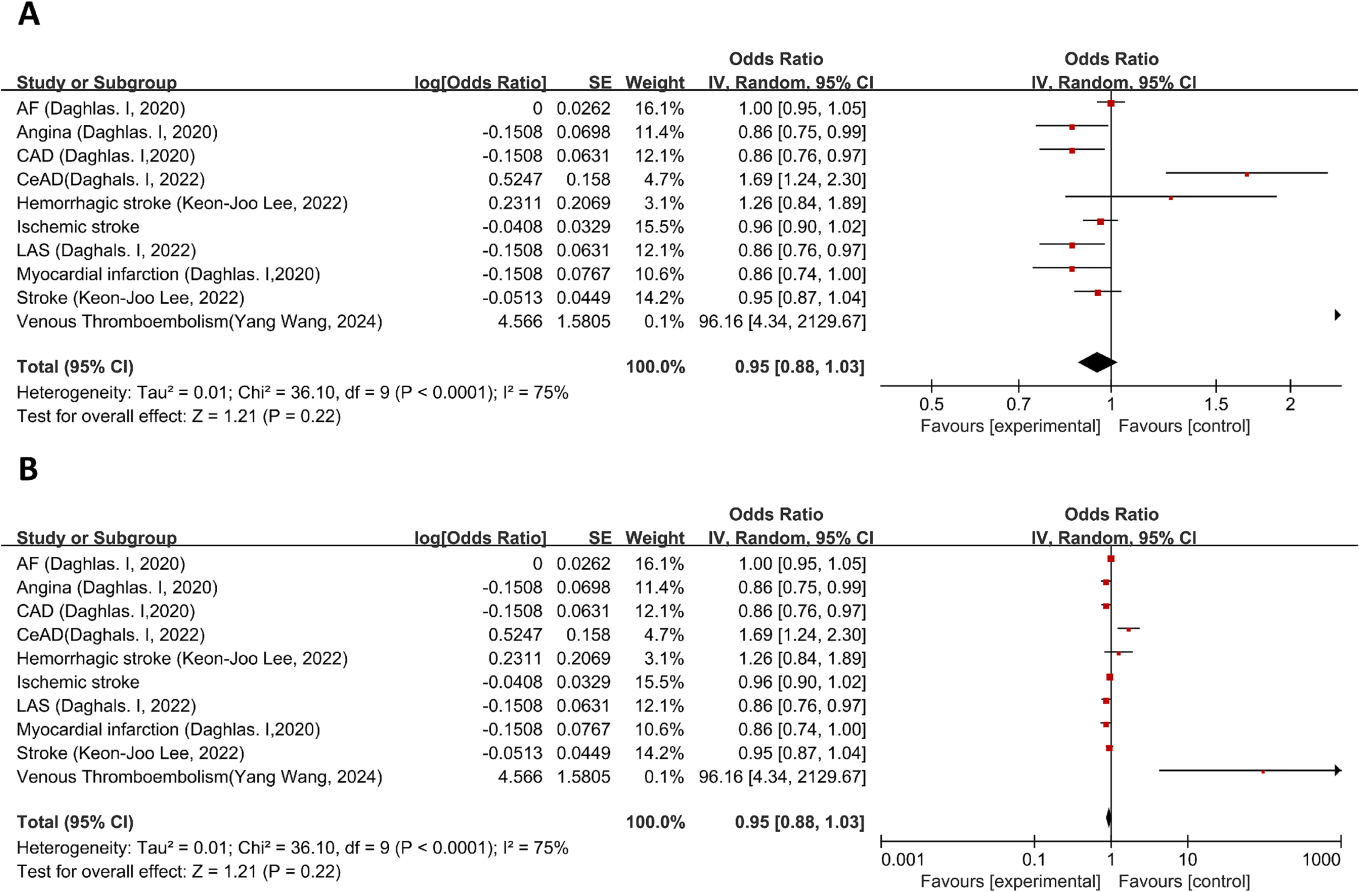
Forward: causal relationship between migraine and diseases of the circulatory system (divided into two parts to show the effect size). **(A)** Narrow vertical axis spacing (to show more details). **(B)** Wide coordinate axis spacing (to show VTE detail). Abbreviations: AF, atrial fibrillation; CAD, coronary artery disease; CeAD, cervical artery dissection; LAS, large artery stroke.

Six studies (17–22) discussed the causal relationship between migraine and diseases of the circulatory system. Mei-Jun Shu et al. (19), Mengmeng Wang et al. (20), and Keon-Joo Lee et al. (18) have done an MR analysis on the causal relationship between migraine and ischemic stroke. Since the data sources of the three studies were different, the three sets of data were meta-analyzed first, and then a second meta-analysis was performed in this study. The MR analysis showed that there was no significant association between migraine and atrial fibrillation (AF) (OR = 1.00, 95% CI: 0.95–1.05), suggesting a possible negative correlation between migraine and angina (OR = 0.86, 95% CI: 0.75–0.99), and coronary artery disease (CAD) showed a similar protective effect (OR = 0.86, 95% CI: 0.76–0.97). Meta-analysis suggested that migraine was a risk factor for cervical artery dissection (CeAD) (OR = 1.69, 95% CI: 1.24–2.30); there was no significant relationship between migraine and hemorrhagic stroke (OR = 1.26, 95%CI: 0.84–1.89) or ischemic stroke (OR = 0.96, 95%CI: 0.90–1.02). The results suggested that there was a negative correlation between migraine and large artery stroke (LAS) (OR = 0.86, 95%CI: 0.76–0.97) and no significant relationship between migraine and myocardial infarction (OR = 0.86, 95%CI: 0.74–1.00) (Figure 2A). The meta-analysis suggested that migraine was a risk factor for venous thromboembolism (VTE) (OR = 96.16, 95% CI: 4.34–2129.67). This estimate was derived from 11 SNPs included in the VTE dataset, with individual SNP F-statistics ranging from 29.76 to 96.77 (all >10), indicating minimal risk of weak instrument bias. Cochran’s Q test for MR-Egger regression and IVW method yielded statistics of 5.610 and 5.973, respectively (both *p* > 0.05), suggesting no significant heterogeneity among SNPs. MR-Egger regression showed the intercept term was not statistically different from zero (*p* = 0.5617), indicating no evidence of genetic pleiotropy. Leave-one-out analysis further confirmed that excluding any single SNP did not substantially alter the causal effect estimate, supporting the robustness of this result. Pooled analysis showed no significant causal association between migraine and diseases of the circulatory system (OR = 0.95, 95% CI: 0.88–1.03, *p* = 0.22). I2 = 75%, suggesting severe heterogeneity. However, migraine is a highly associated risk factor for VTE, this result has been validated by methodology (Figure 2B). For details, see Figure 2.

#### 3.4.2 Sleep–wake disorders

Two studies (23, 24) discussed the causal relationship between migraine and sleep–wake disorders. No significant causal relationship between migraine and insomnia was detected in the Chu, S et al. study (24) (OR = 1.01, 95% CI: 1.00–1.02, *p* = 0.159). There was no evidence in the Daghlas, I et al. (23) of a causal relationship between migraine susceptibility and difficulty awakening (*β* = 0.00, 95% CI: −0.01–0.01, *p* = 0.75) or insomnia (*β* = 0.02, 95% CI: −0.00–0.05, *p* = 0.09).

#### 3.4.3 Diseases of the digestive system

**Figure 3:**
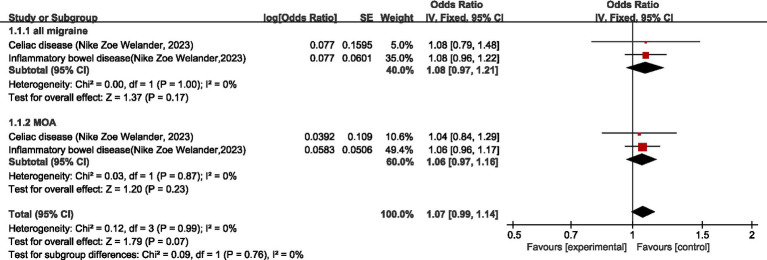
Forward: causal relationship between migraine and diseases of the digestive system.

One study (25) discussed the causal relationship between migraine and disease of digestive system. For all migraine, meta-analysis showed no significant causal relationship between migraine and celiac disease and inflammatory bowel disease (OR = 1.08, 95% CI: 0.97–1.21, *p* = 0.17). For MOA, meta-analysis showed no significant causal relationship between MOA and celiac disease and inflammatory bowel disease (OR = 1.06; 95% CI: 0.97–1.16; *p* = 0.23). Pooled analysis showed no significant causal relationship between migraine and disease of digestive system (OR = 1.07, 95% CI: 0.99–1.14, *p* = 0.07). I2 = 0%, no heterogeneity (see Figure 3).

#### 3.4.4 Diseases of the skin

One study (26) discussed the causal relationship between migraine and the disease of the skin. Guanglu Li et al. (26) did not support a causal relationship between the risk of migraine and its subtypes and psoriasis (migraine-psoriasis: OR = 1.0033, 95% CI: 0.8831–1.1398, *p* = 0.76; MWA-psoriasis: OR = 1.0213, 95% CI: 0.9499–1.0980, *p* = 0.56; MOA-psoriasis: OR = 1.1057, 95% CI: 0.9938–1.2303, *p* = 0.06).

#### 3.4.5 Endocrine, nutritional or metabolic diseases

**Figure 4:**
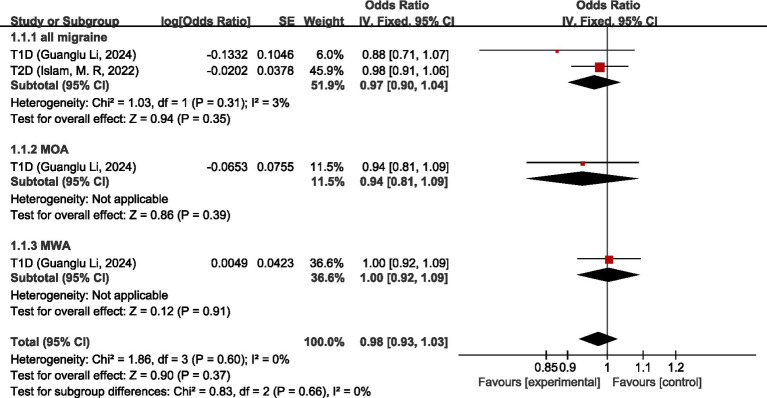
Forward: causal relationship between migraine and endocrine, nutritional or metabolic diseases. T1D, type 1 diabetes; T2D, type 2 diabetes.

Two studies (26, 27) discussed the causal relationship between migraine and endocrine, nutritional or metabolic diseases. Guanglu Li et al. (26) analyzed the MR data from three GWAS data sources (International Headache Genetic Consortium (IHGC), UK Biobank (UKB), and Finnish Genome Study (FinnGen)), respectively. In this paper, the data from these three data sources were meta-analyzed first before analyzing the data during the analysis process. For all migraine, meta-analysis showed that there was no significant causal relationship between migraine and type 1 diabetes (T1D) and type 2 diabetes (T2D) (OR = 0.97, 95% CI: 0.90–1.04, *p* = 0.35). For MOA and MWA, MR analysis showed that neither MOA nor MWA had a significant causal relationship with T1D (OR = 0.94, 95%CI: 0.81–1.09) (OR = 1.00, 95%CI: 0.92–1.09). Pooled analysis showed no significant causal relationship between migraine and endocrine, nutritional or metabolic diseases (OR = 0.98, 95% CI: 0.93–1.03, *p* = 0.37). I2 = 0%, no heterogeneity. See Figure 4 for details.

#### 3.4.6 Diseases of the nervous system

**Figure 5:**
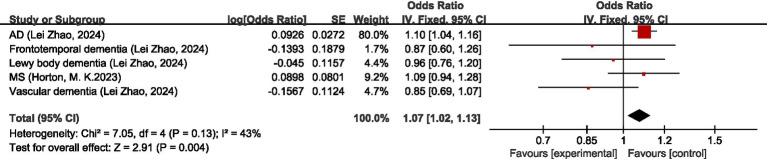
Forward: causal relationship between migraine and diseases of the nervous system. AD, Alzheimer’s disease; MS, multiple sclerosis.

Seven studies (28–34) discussed the causal relationship between migraine and diseases of the nervous system. Chengfeng Xu et al. (32), Hua Xue et al. (30), Daghlas, I et al. (28), Geng, C et al. (33), Lei Zhao et al. (34), and Baranova, A. et al. (31) have studied the causal relationship between migraine and Alzheimer’s disease (AD), and since the GWAS data sources are the same, the newest and the study with the largest sample size, i.e., the study by Lei Zhao et al. (34), was selected for the analysis of our study. The results of the MR analysis showed that there was a significant positive correlation between migraine and AD (OR = 1.01; 95% CI: 1.04–1.16). There was no significant causal relationship between migraine and frontotemporal dementia, Lewy body dementia, multiple sclerosis (MS), or vascular dementia (OR = 0.87, 95% CI: 0.60–1.26) (OR = 0.96, 95% CI: 0.76–1.20) (OR = 1.09, 95% CI: 0.94–1.28) (OR = 0.85, 95% CI: 0.69–1.07). In the overall analysis, there was no significant causal relationship between migraine and disorders of the nervous system (OR = 1.07, 95% CI: 1.02–1.13, *p* = 0.004). I2 = 3%, suggesting mild heterogeneity. For details, see Figure 5.

#### 3.4.7 Neoplasms

One study (35) discussed the causal relationship between migraine and neoplasms. MR in Fang, T et al. (35) showed that migraine was a risk factor for breast cancer (OR = 1.072, 95% CI: 1.035–1.110, *p* = 8.78 × 10^−5^); MOA was associated with an increase in breast cancer risk (OR = 1.042, 95% CI: 1.005–1.081, *p* = 0.0267); MWA was not causally associated with breast cancer (OR = 0.922, 95% CI: 0.840–1.103, *p* = 0.0919).

#### 3.4.8 Diseases of the immune system

Two studies (26, 36) discussed the causal relationship between migraine and diseases of the immune system. Hao Lv et al. (36) and Guanglu Li et al. (26) both investigated the causal relationship between migraine/MOA/MWA and allergic rhinitis (AR). Due to the different data sources of GWAS, the original data were first meta-analyzed before being used for analysis in our study.

**Figure 6:**
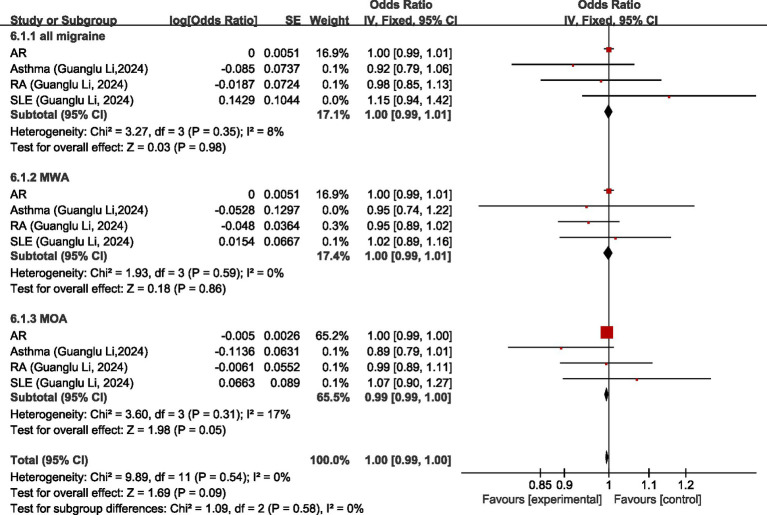
Forward: causal relationship between migraine and diseases of the immune system. AR, allergic rhinitis; RA, rheumatoid arthritis; SLE, systemic lupus erythematosus.

Regarding all migraine, MR analysis showed that there was no significant causal association between all migraine and AR, asthma, rheumatoid arthritis (RA), and systemic lupus erythematosus (SLE), and all migraine was not significantly causally associated with diseases of the immune system (OR = 1.00, 95% CI 0.99–1.01, *p* = 0.98). Regarding MWA, MR analysis showed that there was no significant causal association between MWA and AR, asthma, RA, and SLE, and there was no significant causal association between MWA and diseases of the immune system (OR = 1.00, 95% CI: 0.99–1.01, *p* = 0.86). Regarding MOA, MR analysis showed no significant causal association between MOA and AR, asthma, RA, and SLE, and no significant causal association between MOA and diseases of the immune system (OR = 0.99, 95% CI: 0.99–1.00, *p* = 0.05). Pooled analysis showed no causal relationship between migraine and its subtypes and disorders of the immune system (OR = 1.00, 95%CI: 0.99–1.00, *p* = 0.09); I2 = 0%, no heterogeneity. For details, see Figure 6.

#### 3.4.9 Diseases of the respiratory system

One study (26) discussed the causal relationship between migraine and diseases of the respiratory system. Guanglu Li et al. (26) did not find a significant causal relationship between migraine or its subtypes and asthma (all migraine-asthma: OR = 0.9185, 95% CI: 0.7949–1.0613, *p* = 0.22) (MOA-asthma: OR = 0.8926, 95% CI: 0.7888–1.0101, *p* = 0.07) (MWA-asthma: OR = 0.9486, 95% CI: 0.7357–1.2230, *p* = 0.68).

#### 3.4.10 Diseases of the ear or mastoid process

One study (37) discussed the causal relationship between migraine and diseases of the ear or mastoid process. Kangjia Zhang et al. (37) demonstrated that there was no significant causal relationship between migraine and Meniere’s disease (MD) risk (*p* = 0.825).

#### 3.4.11 Diseases of the genitourinary system

Two studies (38, 39) discussed the causal relationship between migraine and diseases of the genitourinary system. Emmanuel O. Adewuyi et al. (38) found no evidence of a causal relationship between migraine and endometriosis in their study (data not shown in the original article). Wenqiang Zhang et al. (39) showed that genetic susceptibility to migraine does not affect chronic kidney disease (CKD) risk (OR = 1.03, 95% CI = 0.98–1.09; *p* = 0.28).

#### 3.4.12 Other ICD-11 classified diseases

One study (40) discussed the causal relationship between migraine and other ICD-11 classified diseases. Zhen-Ni Zhao et al. (40) did not find a causal relationship between migraine and periodontitis (PD) (OR = 1.00, 95%CI: 0.99–1.00, *p* = 0.65).

#### 3.4.13 Non-disease

Eleven studies (28, 41–50) discussed the causal relationship between migraine and non-disease factors.

In terms of dietary intake non-disease factors, Xinhui Liu et al. (48) showed that migraine on overall alcohol intake (*β* = −0.0571, 95% CI: −0.07, −0.04) as a positive association.

**Figure 7:**
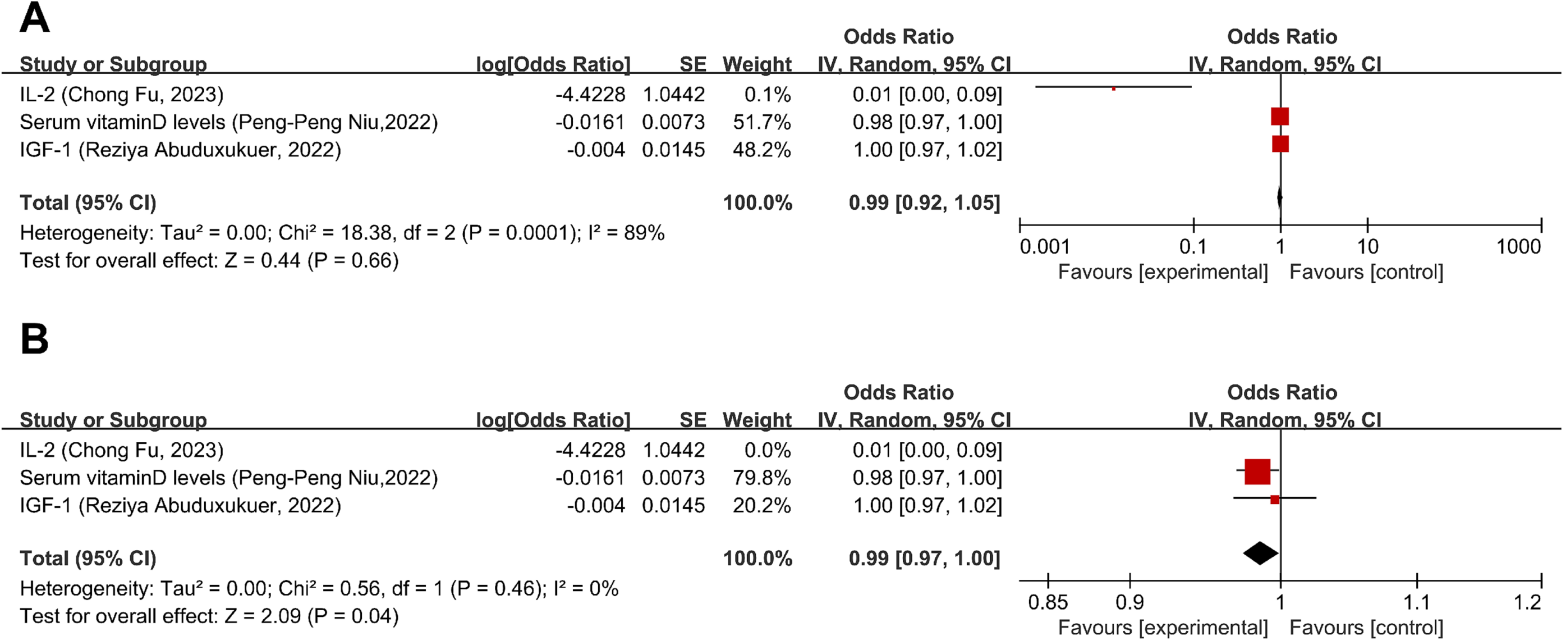
Forward: causal relationship between migraine and physiologic non-disease factors. **(A)** IL-2 included. **(B)** IL-2 eliminated. IGF-1, insulin-like growth factor 1; IL-2, interleukin-2.

Three studies (42–44) discussed the causal relationship between migraine and physiologic non-disease factors. MR analysis showed no significant causal relationship between migraine and insulin-like growth factor 1 (IGF-1) levels or higher serum vitamin D levels (OR = 1.00, 95% CI: 0.97–1.02) (OR = 0.98, 95% CI: 0.97–1.00); there is a heightened risk of migraines and diminished levels of interleukin-2 (IL-2) levels (OR = 0.01, 95% CI: 0.00–0.09) (Figure 7A). Overall, there was no significant causal relationship between migraine and physiologic non-disease factors (OR = 0.99, 95% CI: 0.92–1.05, *p* = 0.66) (Figure 7B). I2 = 89%, suggesting severe heterogeneity and was entirely attributable to the IL-2 data point, as evidenced by the elimination of heterogeneity upon its exclusion (Figure 7B). However, the overall effect remained non-significant after exclusion, as detailed in Figure 7.

Additionally, both Daghlas, I et al. (28) and Brittany L Mitchel et al. (41) studied the relationship between migraine and intracranial volume (ICV); as the GWAS data source was the same, the most recent and largest study containing sample size was selected, i.e., the Brittany L Mitchel et al. (41) to conduct the study analyzed herein, which did not find a significant causal relationship between migraine and ICV (OR: 0.95, 95% CI: 0.89–1.02, *p* = 0.16). Lei Zhao et al. (47) demonstrated that migraine exhibited significant causal effects on two white matter (WM) imaging-derived phenotypes (IDPs) (both the mode of anisotropy of the right uncinate fasciculus and the orientation dispersion index of the left superior cerebellar peduncle decreased) (*p* < 3.29 × 10^−4^). Huo, J et al. (45) found no association between migraine and white matter lesions (*p* > 0.05). Xiangyue Meng et al. (50), He, Q et al. (46), and Kang Qu et al. (49) all investigated the causal relationship between migraine and gut microbiota. Since the GWAS data sources were the same, the most recent study with the largest sample size, Kang Qu et al. (49), was chosen for the analysis of our study. The study did not find a significant causal relationship between migraine and gut microbiota.

### 3.5 Causal relationship between multiple diseases and migraine

#### 3.5.1 Mental, behavioral or neurodevelopmental disorders

**Figure 8:**
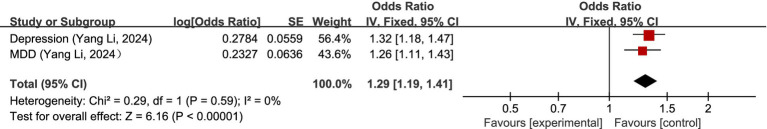
Reverse: causal relationship between mental, behavioral or neurodevelopmental disorders and migraine. MDD, major depressive disorder.

Two studies (51, 52) discussed the causal relationship between mental, behavioral or neurodevelopmental disorders and migraine. MR analysis showed a significant causal relationship between depression and migraine (OR = 1.32, 95% CI: 1.18–1.47). Yang Li et al. (52) and Xiaofeng Lv et al. (51) both investigated the causal relationship between major depressive disorders (MDD) and migraine. Since the GWAS data source was the same, the most recent study with the largest sample size, i.e., Yang Li et al. (52), was chosen for the analysis of our study. The results of the MR analysis showed that there was also a significant causal relationship between major depressive disorder and migraine (OR = 1.26, 95% CI: 1.11–1.43). Overall, there was a positive correlation between mental, behavioral or neurodevelopmental disorders and migraine (OR = 1.29, 95% CI: 1.19–1.47, *p* < 0.05). I2 = 0%, no heterogeneity. For details, see Figure 8.

#### 3.5.2 Disease of the circulatory system

Two studies (22, 53) discussed the causal relationship between disorders of the circulatory system and migraine. Xu-Peng Wu et al. (53) showed that VTE was associated with an increased risk of MWA (OR = 1.137, 95% CI: 1.062–1.218, *p* = 2.47 × 10^−4^). Yang Wang et al. (22) showed that VTE was a risk factor for migraine (OR = 1.002, 95% CI: 1.000–1.004, *p* = 0.016).

#### 3.5.3 Sleep–wake disorders

**Figure 9:**
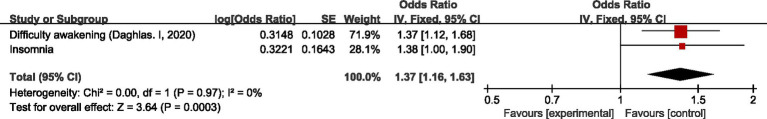
Reverse: causal relationship between sleep–wake disorders and migraine.

Three studies (23, 24, 52) discussed the causal relationship between sleep–wake disorders and migraine. Yang Li et al. (52) and Chu S et al. (24) both did MR on the causal relationship between insomnia and migraine. Due to the difference in data sources between the two studies, the original data were meta-analyzed first before the meta-analysis of our study. MR analysis showed that there was a positive correlation between difficulty awakening and migraine (OR = 1.37, 95%CI: 1.12–1.68); meta-analysis showed insomnia had no significant causal relationship with migraine (OR = 1.38, 95%CI: 1.00–1.90). Pooled analysis showed a positive correlation between sleep–wake disorders and migraine (OR = 1.37, 95% CI: 1.16–1.63, *p* < 0.05). I2 = 0%, no heterogeneity. See Figure 9 for details.

#### 3.5.4 Diseases of the digestive system

**Figure 10:**
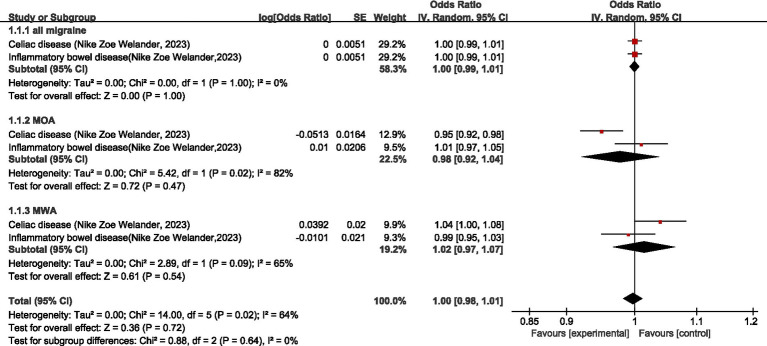
Reverse: causal relationship between diseases of the digestive system and migraine.

One study (25) discussed the causal relationship between diseases of the digestive system and migraine. Regarding all migraine, meta-analysis showed that there was no significant causal relationship between diseases of the digestive system and all migraine (OR = 1.00, 95% CI: 0.99–1.01). Regarding MOA, MR analysis showed a negative association between celiac disease and MOA (OR = 0.95, 95% CI: 0.92–0.98); no significant causal association between inflammatory bowel disease and MOA (OR = 1.01, 95% CI: 0.97–1.05); and no significant causal relationship between diseases of the digestive system and MOA (OR = 0.98, 95% CI: 0.92–1.04, *p* = 0.47). Regarding MWA, MR analysis showed a positive association between celiac disease and MWA (OR = 1.04, 95% CI: 1.00–1.08); there was no significant causal association between inflammatory bowel disease and MWA (OR = 0.99, 95% CI: 0.95–1.03), and the association between the digestive system and MWA was not significant (OR = 0.98, 95% CI: 0.92–1.04). In the combined analysis, there was no significant causal relationship between diseases of the digestive system and migraine (OR = 1.00, 95% CI: 0.98–1.01, *p* = 0.72). I2 = 64%, moderate heterogeneity. See Figure 10 for details.

#### 3.5.5 Diseases of the skin

**Figure 11:**
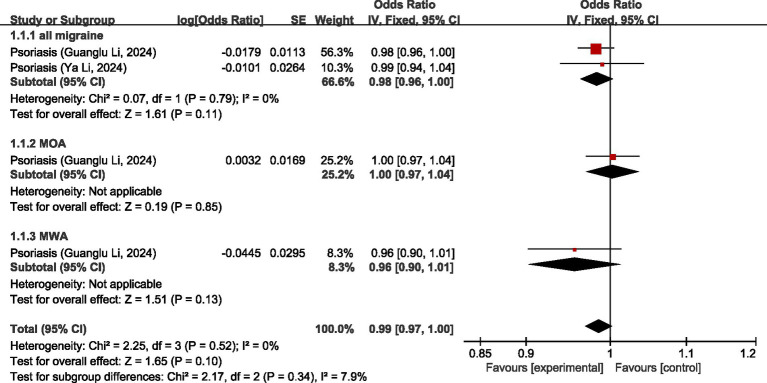
Reverse: causal relationship between diseases of the skin and migraine.

One study (26) discussed the causal relationship between diseases of the skin and migraine. In the study by Guanglu Li et al. (26), MR analysis was performed on data from three GWAS data sources (IHGC, UKB, FinnGen), respectively. We meta-analyzed the data from these three GWAS data sources before analyzing the data in our study. The results of MR analysis showed that, regarding all migraine, there was no significant relationship between psoriasis and all migraine (OR = 0.98, 95% CI: 0.96–1.00, *p* = 0.11). Regarding MOA and MWA, there was no significant causal relationship between psoriasis and both MOA and MWA (OR = 1.00, 95% CI: 0.97–1.04, *p* = 0.85) (OR = 0.96, 95% CI: 0.90–1.01, *p* = 0.13). Pooled analyses showed there was no significant association between diseases of the skin and migraine (OR = 0.99, 95% CI: 0.97–1.00, *p* = 0.10). I2 = 7.9%, mild heterogeneity. See Figure 11 for details.

#### 3.5.6 Endocrine, nutritional or metabolic diseases

**Figure 12:**
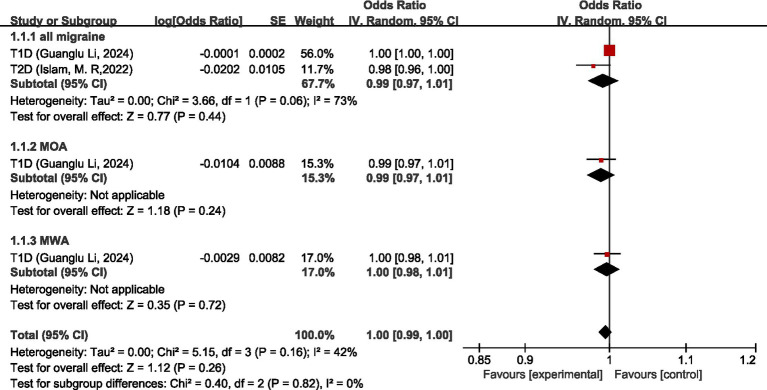
Reverse: causal relationship between endocrine, nutritional or metabolic diseases and migraine. T1D, type 1 diabetes; T2D, type 2 diabetes.

Two studies (26, 27) discussed the causal relationship between endocrine, nutritional or metabolic diseases and migraine. In the study by Guanglu Li et al. (26), MR analysis was performed on data from three GWAS data sources (IHGC, UKB, FinnGen), respectively. We meta-analyzed the data from these three GWAS data sources before analyzing the data in our study. For all migraine, MR analysis showed that there was no significant causal relationship between T1D and T2D (OR = 0.99, 95% CI: 0.97–1.01, *p* = 0.44). For MOA and MWA, MR analysis showed that there was no significant causal relationship between T1D and both MOA and MWA (OR = 0.99, 95%CI: 0.97–1.01) (OR = 1.00, 95%CI: 0.98–1.01). Pooled analysis showed no significant causal association between endocrine, nutritional or metabolic diseases and migraine (OR = 1.00, 95% CI: 0.99–1.00, *p* = 0.26). I2 = 42%, mild heterogeneity. See Figure 12 for details.

#### 3.5.7 Diseases of the nervous system

One study (33) discussed the causal relationship between disorders of the nervous system and migraine. Geng, C et al. (33) showed no significant causal relationship between AD and migraine (OR = 1.000, 95%CI: 0.999–1.006, *p* = 0.971).

#### 3.5.8 Diseases of the immune system

Four studies (26, 36, 54, 55) discussed the causal relationship between diseases of the immune system and migraine. Both Guanglu Li et al. (26) and Danfeng Xu et al. (54) investigated the causal relationship between SLE and migraine, MOA, or MWA; and Meixuan Ren et al. (55) investigated the causal relationship of SLE with MOA or MWA. Due to the different GWAS data sources, the original data were meta-analyzed before analyzing in our study (for all migraine, meta-analyzed Guanglu Li et al. (26) and Danfeng Xu et al. (54); for MOA and MWA, meta-analyzed Guanglu Li et al. (26), Danfeng Xu et al. (54), and Meixuan Ren et al. (55)). Both Guanglu Li et al. (26) and Hao Lv et al. (36) investigated the causal relationship between AR and migraine and its subtypes, and because of the different data sources of GWAS, the original data were meta-analyzed first and then analyzed in our study.

**Figure 13:**
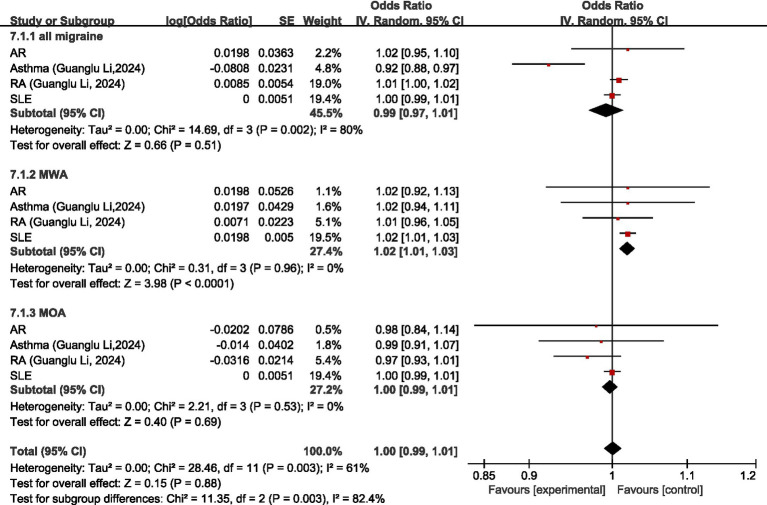
Reverse: causal relationship between diseases of the immune system and migraine. AR, allergic rhinitis; RA, rheumatoid arthritis; SLE, systemic lupus erythematosus.

Regarding all migraine, MR analysis showed that AR, RA, and SLE had no significant causal relationship with migraine, asthma was a protective factor for all migraine (OR = 0.92, 95% CI: 0.88–0.97); meta-analysis showed diseases of the immune system were not significantly causally related to all migraine (OR = 0.99, 95% CI: 0.97–1.01, *p* = 0.51). Regarding MWA, MR analysis showed that AR, asthma, and RA had no significant causal relationship with MWA; SLE was a risk factor for MWA (OR = 1.02, 95%CI: 1.01–1.03); meta-analysis showed that diseases of the immune system had a positive correlation with MWA (OR = 1.02, 95%CI: 1.01–1.03, *p* < 0.05). Regarding MOA, MR analysis showed that AR, asthma, RA, and SLE had no significant causal association with MOA, and diseases of the immune system had no significant causal relationship with MOA (OR = 1.00, 95% CI: 0.99–1.01, *p* = 0.69). Comprehensive meta-analysis showed no significant causal relationship between diseases of the immune system and migraine (OR = 1.00, 95% CI: 0.99–1.01, *p* = 0.88). I2 = 61%, moderate heterogeneity. See Figure 13 for details.

#### 3.5.9 Diseases of the respiratory system

One study (26) discussed the causal relationship between disorders of the respiratory system and migraine. Guanglu Li et al. (26) did not find a significant causal relationship between asthma and migraine and its subtypes (asthma-all migraine: OR = 0.9224, 95%CI: 0.8816–1.0175, *p* = 0.16) (asthma-MOA: OR = 0.9861, 95%CI: 0.9113–1.0671, *p* = 0.72) (asthma-MWA: OR = 1.0199, 95% CI: 0.9376–1.1093, *p* = 0.64).

#### 3.5.10 Diseases of the ear or mastoid process

One study (37) discussed the causal relationship between diseases of the ear or mastoid process and migraine. Kangjia Zhang et al. (37) demonstrated no significant causal relationship between migraine and MD risk (OR = 0.999, *p* = 0.020).

#### 3.5.11 Diseases of the genitourinary system

**Figure 14:**
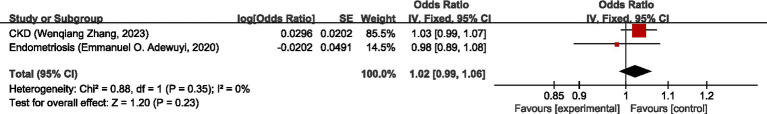
Reverse: causal relationship between diseases of the genitourinary system and migraine. CKD, chronic kidney disease.

Two studies (38, 39) discussed the causal relationship between diseases of the genitourinary system and migraine. MR analysis showed that there was no significant causal relationship between CKD and migraine (OR = 1.03, 95% CI: 0.99–1.07), or endometriosis and migraine (OR = 0.98, 95% CI: 0.89–1.08). Pooled meta-analysis showed no significant causal relationship between diseases of the genitourinary system and migraine (OR = 1.02, 95%CI: 0.99–1.06, *p* = 0.23). I2 = 0%, no heterogeneity. For details, see Figure 14.

#### 3.5.12 Other ICD-11 classified diseases

Zhen-Ni Zhao et al. (40) found no significant causal relationship between PD and migraine (OR = 1.000, 95% CI: 0.99–1.00, *p* = 0.65).

#### 3.5.13 Non-disease

Twenty-six studies (41–50, 52, 56–70) discussed the causal relationship between non-disease factors and migraine.

**Figure 15:**
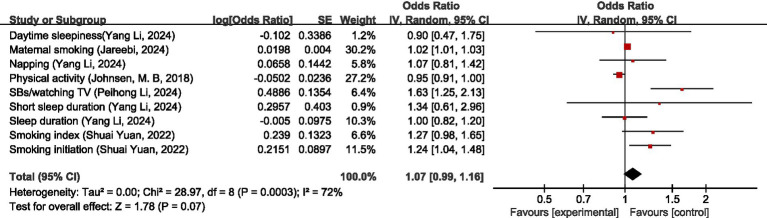
Reverse: causal relationship between behavioral habits non-disease factors and migraine. SBs, sedentary behaviors.

For behavioral habits non-disease factors, MR analysis showed that the four sleep habits of daytime sleeping, napping, short sleep duration, and sleep duration had no significant causal relationship with migraine; maternal smoking was a risk factor for migraine (OR = 1.02, 95% CI: 1.01–1.03), smoking index had no significant causal relationship with migraine (OR = 1.27, 95%CI: 0.98–1.65), smoking initiation was a risk factor for migraine (OR = 1.24, 95%CI: 1.04–1.48); physical activity had no significant causal relationship with migraine (OR = 0.95, 95% CI: 0.91–1.00); watching TV, a sedentary behavior (SBs), was a risk factor for migraine (OR = 1.63, 95% CI: 1.25–2.13). Pooled MR analysis showed that behavioral habits non-disease factors were not significantly causally associated with migraine (OR = 1.07, 95%CI: 0.99–1.16, *p* = 0.07), I2 = 72%, moderate heterogeneity. See Figure 15 for details.

Regarding dietary intake non-disease factors, Shuai Yuan et al. (61), Chen, H et al. (60), Jareebi et al. (66), and Jinjin Zhang et al. (67) studied the causal relationship between coffee consumption and all migraine. Since the data sources were the same, the most recent study with the largest sample size, i.e., Jinjin Zhang et al. (67), was chosen for the analysis in our study; Chen, H et al. (60) and Jinjin Zhang et al. (67) both studied the causal relationship between coffee intake and MWA. Because of the same data source, the most recent study with the largest sample size, Jinjin Zhang et al. (67), was chosen for the analysis of our study.

**Figure 16:**
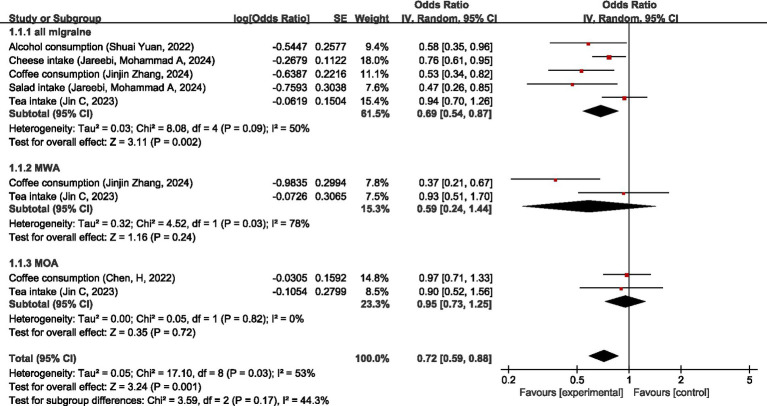
Reverse: causal relationship between dietary intake non-disease factors and migraine.

MR analysis showed that for all migraine, tea intake was not significantly causally associated with all migraines (OR = 0.94, 95% CI: 0.70–1.26), and higher alcohol consumption, cheese intake, coffee consumption, and salad intake were all protective factors for all migraines (OR = 0.58, 95% CI: 0.35–0.96), (OR = 0.76, 95% CI: 0.61–9.85), (OR = 0.53, 95% CI: 0.34–0.82), and (OR = 0.47, 95% CI: 0.26–0.85). Overall, these dietary intake non-disease factors were protective factors for all migraines (OR = 0.69, 95% CI: 0.54–0.87, *p* < 0.05). For MWA, tea intake was not significantly causally associated with MWA (OR = 0.93, 95% CI: 0.51–1.70), and higher coffee consumption was a protective factor for MWA (OR = 0.37, 95% CI: 0.21–0.67); these dietary intake non-disease factors have no association with MWA (OR = 0.59, 95% CI: 0.24–1.44, *p* = 0.24). For MOA, both coffee consumption and tea intake were not significantly causally associated with MOA (OR = 0.97, 95%CI: 0.71–1.33) (OR = 0.90, 95%CI: 0.52–1.56); these dietary intake non-disease factors were not significantly causally associated with MOA (OR = 0.95, 95%CI: 0.73–1.25, *p* = 0.72). Pooled MR analysis showed no significant causal relationship between dietary intake non-disease factors and migraine (OR = 0.72, 95%CI: 0.59–0.88, *p* = 0.001). I2 = 53%, suggesting moderate heterogeneity. For details, see Figure 16.

Furthermore, Xinhui Liu et al. (48) focused on 83 dietary habits; their study showed that more cups of coffee, oily fish, and cheese intake were significantly negatively associated with the risk of migraine (OR = 0.71, 95%CI: 0.59–0.86) (OR = 0.73, 95%CI: 0.59–0.89) (OR = 0.78, 95%CI: 0.63–0.95). This is consistent with the results of this study. They also found that there was an insufficiency of evidence of negative associations between more vegetables (OR = 0.72, 95% CI: 0.57–0.92) as well as wholemeal/wholegrain bread type (OR = 0.76, 95% CI: 0.63–0.92) and migraine. Additionally, they found weak evidence that drinks with meals (OR = 0.61, 95% CI: 0.47–0.80), more red wine (OR = 0.65, 95% CI: 0.51–0.82), and ore alcohol (OR = 0.74, 95% CI: 0.62–0.88) were associated with a decrease in risk of migraine; taking muesli was negatively associated with migraine (OR = 0.65, 95% CI: 0.48–0.89), while cornflakes/frosties were positively associated with migraine (OR = 1.53, 95% CI: 1.14–2.05); more white bread was associated with an increase in risk of migraine; poultry intake was positively associated with migraine (OR = 1.70, 95% CI: 1.19–2.43). Hui Zheng et al. (63) suggested that higher vitamin B12 intake was a protective factor for MWA (OR = 0.49, 95% CI: 0.24–0.99, *p* = 0.046).

Regarding physiologic non-disease factors, MR analysis showed that for all migraine, activated partial thromboplastin time (APTT), average thickness, gray matter volume (GMV), hepatocyte growth factor (HGF), white matter hyperintensities (WMH) had no significant causal relationship with migraine; higher diastolic blood pressure (DBP), pulse pressure (PP), systolic blood pressure (SBP), elevation of serum calcium levels by 1 mg/dL, higher coagulation factor VIII (FVIII) activity, phosphorylated fibrinopeptid A level, and von Willebrand factor (vWF) levels were associated with the increased risk of migraine; higher fibrinogen levels, Hippocampal volume (HV), IGF-1 level, neuralized E3 ubiquitin-protein ligase 1, surface area (cortical) (SA), serum vitamin D levels, and pulse pressure (PP) were associated with the decreased risk of migraine. Overall, there was no significant causal relationship between physiologic non-disease factors and all migraine (OR = 1.02, 95% CI: 0.99–1.04, *p* = 0.29).

For MWA, serum vitamin D levels did not have a significant causal relationship with MWA; FVIII activity, phosphorylated fibrinopeptide A, and vWF levels were risk factors for MWA; higher APTT, fibrinogen level, and IGF-1 were protective factors for MWA. Overall, there was no significant causal relationship between physiologic non-disease factors and MWA (OR = 1.00, 95% CI: 0.91–1.10, *p* = 0.98).

For MOA, IGF-1, and serum vitamin D levels had no significant causal relationship with MOA; higher DBP, PP, and elevation of serum calcium levels by 1 mg/dL was associated with the increased risk of MOA. Overall, there was no significant causal relationship between physiologic non-disease factors and MOA (OR = 1.15, 95% CI: 0.94–1.40, *p* = 0.18).

**Figure 17:**
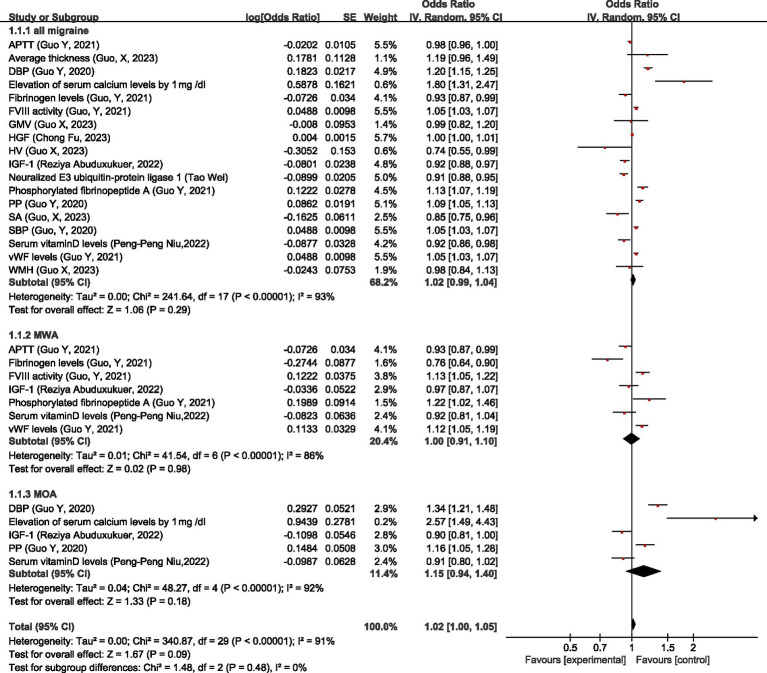
Reverse: causal relationship between physiologic non-disease factors and migraine. APTT, activated partial thromboplastin time; DBP, diastolic blood pressure; FVIII, coagulation factor VIII; GMV, gray matter volume; HGF, hepatocyte growth factor; HV, Hippocampal volume; IGF-1: insulin-like growth factor 1; PP, pulse pressure; SA, surface area (cortical); SBP, systolic blood pressure; vWF, von Willebrand factor; WMH, white matter hyperintensities.

Overall, there is no causal relationship between physiologic non-disease factors and migraine (OR = 1.02, 95% CI: 1.00–1.05, *p* = 0.09), see Figure 17.

Additionally, Brittany L. Mitchel et al. (41) found that there was a negative effect of larger ICV on migraine risk (OR = 0.91, 95%CI: 0.85–0.97, *p* = 0.006). Lei Zhao et al. (47) identified two WM IDPs that exhibited significant causal effects on migraine in the MR analysis (*p* < 3.29e × 10^−4^). Huo, J et al. (45) did not find any effect of white matter lesions (WMLs) on migraine. Xiangyue Meng et al. (50), He, Q et al. (46), and Kang Qu et al. (49) all investigated the relationship between gut microbiota and migraine. Since the GWAS data sources are the same, the most recent study with the largest sample size, i.e., Kang Qu et al. (49), was chosen for the analysis of this study. It was found that only the genus *LachnospiraceaeUCG001* remained significantly associated with migraine (OR = 1.12, 95% CI: 1.05–1.20, *p* = 3.65 × 10^−4^). Kang Qu et al. (68) showed no association between low-density lipoprotein cholesterol (LDL-C), Apolipoprotein B (APOB), total cholesterol (TC) and migraine. Peng-Peng Niu et al. (70) showed that LRP11 (a protein) was significantly associated with the risk of any migraine (OR = 0.968, 95% CI: 0.955–0.981, *p* = 1.27 × 10^−6^) and significantly associated with migraine subtypes. ITIH1 (a protein) was significantly associated with the risk of migraine (OR = 1.044, 95% CI = 1.024–1.065, *p* = 1.08 × 10^−5^). ADGRF5 (a protein) was significantly associated with the risk of migraine (OR = 0.964, 95% CI: 0.946–0.982, *p* = 8.74 × 10^−5^) and suggestively associated with MWA.

Hui Zheng et al. (63) suggested that more years of schooling was negatively associated with MOA (OR = 0.57, 95%CI: 0.44–0.75, *p* < 0.0001), and eicosapentaenoic acid status level (OR = 2.54, 95%CI: 1.03–6.26, *p* = 0.043) was a risk factor for MWA. Guoliang Zhu et al. (65) found there was a causal relationship between delayed AFS and risk for migraine (OR = 0.73, 95% CI: 0.61–0.86), both for MWA (OR = 0.72, 95% CI: 0.58-0.89) and MOA (OR = 0.66, 95% CI:0.51-0.86). Daghlas, I et al. (28) found that genetic liability to migraine was not associated with intelligence (standardized beta = 0.01, 95% CI: 0.00–0.02, *p* = 0.13).

### 3.6 Overview of the establishment of a causal relationship between migraine and multiple factors

Overall, regarding migraine, migraine was a risk factor for 3 diseases (AD, CeAD, and VTE) and a protective factor for 3 diseases (CAD, angina, and LAS), 3 behavioral habits factors (delayed AFS, more physical activity, and maternal smoking), 1 dietary intake factors (more alcohol consumption), 3 physiologic factors (higher IL-2, BMI, and serum vitamin D level). Migraine had no association with 24 diseases (AF, hemorrhagic, ischemic stroke, stroke, myocardial infarction, insomnia, difficulty awakening, celiac disease, inflammatory bowel disease, psoriasis, T1D, T2D, frontotemporal dementia, Lewy body dementia, MS, vascular dementia, AR, asthma, RA, SLE, MD, and PD), 5 physiologic factors (IGF-1, higher serum vitamin D levels, ICV, WMLs, and gut microbiota). Six diseases (VTE, breast cancer, insomnia, difficulty awakening, MDD, and depression), 2 behavioral habits factors (watching TV, smoking initiation), and 11 physiologic factors (higher FVIII activity, vWF levels, phosphorylated fibrinopeptide A level, HGF, SBP, DBP, PP, elevation of calcium level by 1 mg/dL, ITIH1, *SREBF2*, and *LachnospiraceaeUCG001*) were risk factors of migraine. Three behavioral habits factors (delayed AFS, more years of schooling, and physical activity), 4 dietary intake factors (more alcohol consumption, coffee consumption, cheese intake, and salad intake), and 13 physiologic factors (higher fibrinogen levels, APTT, serum vitamin D level, IGF-1, SA, HV, neuralized E3 ubiquitin-protein ligase 1, LRP11, ADGRF5, APO-A1, *REV1*, ICV, and BMI) were protective factors of migraine. Fifteen diseases (insomnia, celiac disease, inflammatory bowel disease, psoriasis, T1D, T2D, AD, AR, RA, SLE, asthma, MD, chronic kidney disease, endometriosis, and PD), 6 behavioral habits factors (daytime sleeping, napping, short sleep duration, sleep duration, smoking index, and physical activity), 1 dietary intake factor (more tea intake), and 8 physiologic factors (APTT, average thickness, GMV, HGF, WMH, LDL-C, APOB, TC) had no association with migraine.

Regarding MOA, MOA was a risk factor for 2 diseases (AD and CeAD), a protective factor for 2 diseases (CAD and LAS), and 1 behavioral habits factor (delayed AFS). MOA had no association with 8 diseases (celiac disease, inflammatory bowel disease, psoriasis, T1D, asthma, AR, SLE, and RA). One disease (breast cancer), 1 behavioral habits factor (watching TV), and 3 physiologic factors (higher DBP, PP, and elevation of serum calcium level by 1 mg/dL) were risk factors of MOA. One disease (celiac disease), 2 behavioral habits factors (delayed AFS and more years of schooling), and 1 physiologic factor (IGF-1) were protective factors of MOA. Seven diseases (inflammatory bowel disease, psoriasis, T1D, AR, asthma, RA, and SLE), 2 dietary intake factors (more coffee consumption and tea intake), and 2 physiologic factors (IGF-1and serum vitamin D level) showed no association with MOA.

**Figure 18:**
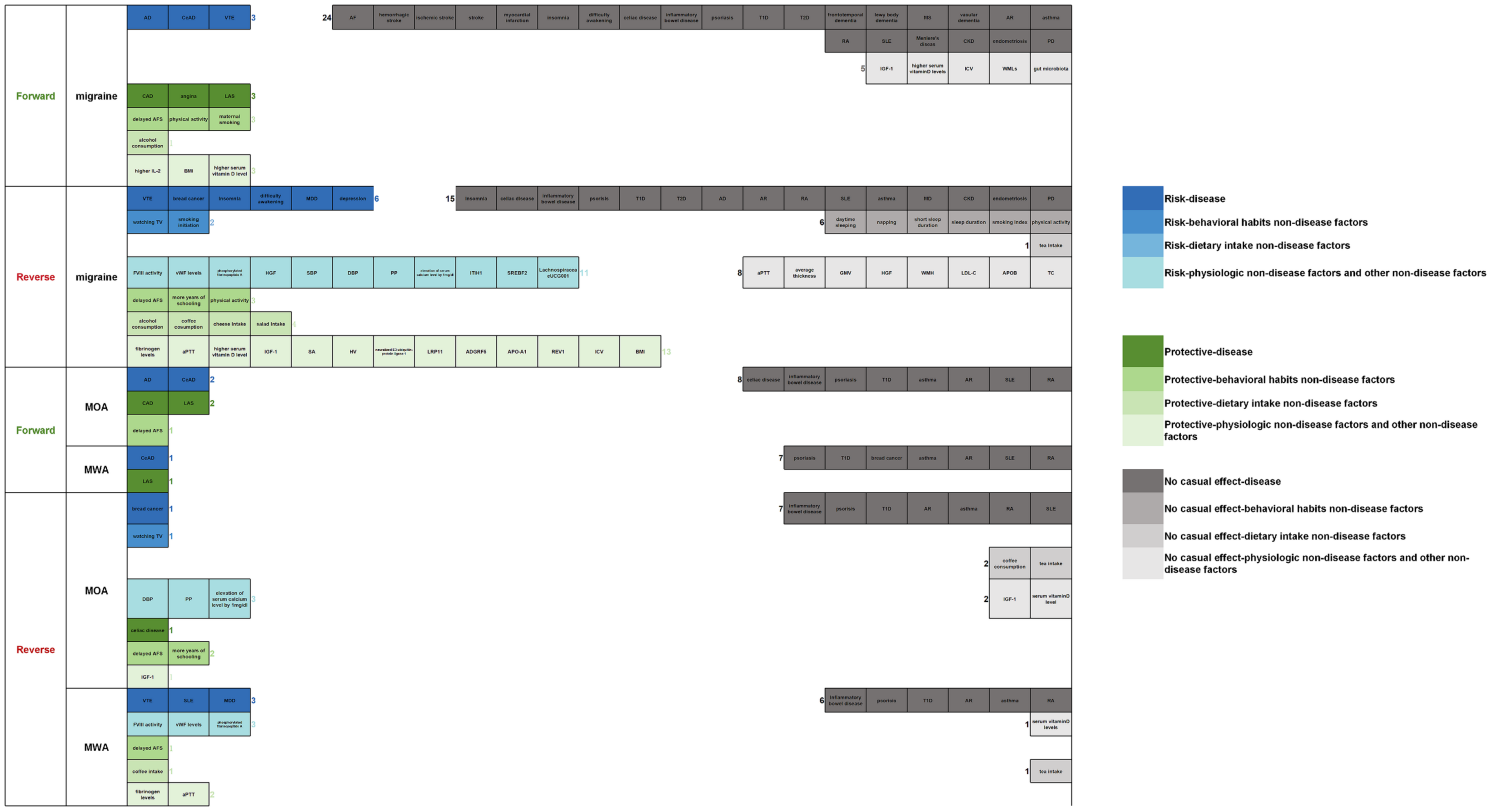
Overview of the establishment of a causal relationship between migraine and multiple factors (forward/reverse).

Regarding MWA, MWA was a risk factor of 1 disease (CeAD) and a protective factor of 1 disease (LAS). MWA had no association with 7 diseases (psoriasis, T1D, breast cancer, asthma, AR, SLE, and RA). Three diseases (VTE, SLE, and MDD) and 3 physiologic factors (higher FVIII activity, vWF levels, and phosphorylated fibrinopeptide A) were risk factors of MWA; 1 behavioral habits factor (delayed AFS), 1 dietary intake factor (more coffee consumption), and 2 physiologic factors (fibrinogen levels and APTT) were protective factors of MWA. Six diseases (inflammatory bowel disease, psoriasis, T1D, AR, asthma, and RA), 1 dietary intake factor (more tea intake), and 1 physiologic factor (higher serum vitamin D levels) showed no association with MWA (see Figure 18).

### 3.7 Relationships between genes associated with migraine or their expression products and drug targets

Seven studies (57, 68, 70–74) have discussed the relationship between genes or their expression products and drug targets associated with migraine. SNP rs1051730 is by far the strongest genetic variant associated with smoking behavior found in genome-wide studies (75, 76). Johnsen, M. B et al. (57) indicated that no association was found between the rs1051703T allele and migraine (all participants: OR = 0.98, 95%CI: 0.95–1.02, *p* = 0.38; never smokers: OR = 0.99, 95%CI: 0.93–1.04, *p* = 0.63; ever smokers: OR = 0.97, 95%CI: 0.92–1.03, *p* = 0.32). Bi Y et al. (71) found that 3-Hydroxy-3-Methylglutaryl-CoA Reductase (*HMGCR*) inhibition, corresponding to the reduction in LDL-C, was significantly causally associated with a lower risk of migraine (OR = 0.73, 95% CI: 0.60–0.89, *p* = 0.0016). Lipoprotein lipase (LPL) enhancement was significantly causally associated with a lower risk of migraine (OR = 0.89, 95% CI: 0.83–0.96, p = 0.0016). This suggests that LPL and *HMGCR* have the potential to serve as candidate drug targets for the treatment or prevention of migraine. Chengcheng Zhang et al. (72) identified 21 druggable genes significantly associated with migraine (*BRPF3, CBFB, CDK4, CHD4, DDIT4, EP300, EPHA5, FGFRL1, FXN,* HMGCR*, HVCN1, KCNK5, MRGPRE, NLGN2, NR1D1, PLXNB1, TGFB1, TGFB3, THRA, TLN1, and TP53*), two of which were significant in both blood and brain (*HMGCR and TGFB3*). *TGFB3* was mainly associated with IGF-1 levels, and *HMGCR* was highly correlated with LDL- C. Hong, P et al. (73) demonstrated that genotypes of *HMGCR* related to higher LDL-C level might increase the risk of migraine (OR = 1.46, 95% CI: 1.03–2.07, *p* = 0.035) and MWA (OR = 2.03, 95% CI: 1.20–3.42, *p* = 0.008), genotypes of APOB related to higher LDL-C level might decrease the risk of MOA (OR = 0.62, 95% CI: 0.47–0.81, *p* = 0.000), and genotypes of *PCSK9* related to higher LDL-C level might decrease the risk of migraine (OR = 0.75, 95% CI: 0.64–0.89, p = 0.001) and MWA (OR = 0.69, 95% CI: 0.54–0.89, *p* = 0.004). Kang Qu et al. (68) indicated that *HMGCR* expression (OR = 1.55, 95% CI: 1.30–1.84, *p* = 6.87 × 10^−7^) and the circulating levels of three lipids (LDL-C, APOB, and TC) adjusted by *HMGCR* expression (OR = 1.55, 95% CI: 1.30–1.84, p = 6.87 × 10^−7^) were significantly associated with an increased risk of migraine (LDL-C: OR = 1.51, 95% CI 1.21–1.88, *p* = 2.50 × 10^−4^; APOB: OR = 2.12, 95% CI: 1.56–2.87, *p* = 1.35 × 10^−6^; TC: OR = 1.63, 95% CI: 1.30–2.06, *p* = 2.93 × 10^−5^). Four groups of studies (68, 71–73) on the relationship between *HMGCR* and migraine reached consistent conclusions, and these findings indicate a correlation between HMGCR and migraine. Future investment in research on HMGCR inhibitors may provide a new approach to migraine prevention.

Jianxiong Gui et al. (74) showed that *REV1* may reduce the migraine risk by regulating DNA damage repair, while *SREBF2* may increase the risk of migraine by regulating cholesterol metabolism. The *REV1* gene is located on chromosome 2q11.2, and MR analysis confirmed a causal relationship between *REV1* and migraine (*p* < 0.05). MR analysis of testicular tissue confirmed a significant causal relationship between the *SREBF2* gene and migraine (OR = 1.10, 95% CI: 1.01–1.19, p < 0.05). Pengpeng Niu et al. (70) found that LRP11 was significantly associated with the risk of migraine (OR = 0.968, 95% CI: 0.955–0.981, *p* = 1.27 × 10^−6^). ITIH1 was significantly associated with the risk of migraine (OR = 1.044, 95% CI: 1.024–1.065, *p* = 1.08 × 10^−5^). ADGRF5 was significantly associated with the risk of migraine (OR = 0.964, 95% CI: 0.946–0.982, *p* = 8.74 × 10^−5^).

## 4 Discussion

Migraine is a chronic, progressive neurological disorder (77). So far, a large number of studies have been conducted on migraine and its causal factors, but almost no one has yet integrated these results. This is a systematic review focusing on evidence concerning factors contributing to migraine from Mendelian randomization studies.

A review of the pathogenesis of migraine found that three theories—cortical spreading depression (CSD), trigeminal vascular theory, and genetics—are widely accepted in academia. The review by Pleș H et al. further highlighted the key role of calcitonin gene-related peptide (CGRP) signaling in migraine pathophysiology (78). However, despite significant research, the precise biological mechanisms linking these established pathways (CSD, trigeminovascular activation, CGRP) and emerging factors to migraine onset and progression remain incompletely understood. This mechanistic uncertainty poses major challenges for treatment, often resulting in suboptimal outcomes, recurrence, and significant impacts on patients’ socialization and quality of life. In this study, we comprehensively integrated evidence of causal associations between migraine and multidimensional exposure factors (including disease, non-disease, and genetic factors, both forward and reverse) through a Mendelian randomization systematic review. Crucially, these MR-derived causal relationships provide new avenues to explore the underlying biological mechanisms. By identifying robust causal factors, this work generates specific hypotheses for how these factors might interact with or modulate known pathways (like CGRP signaling) or implicate novel biological processes in migraine pathogenesis, thereby offering direction for future mechanistic research and therapeutic development.

This systematic review included a total of 60 MR studies, comprising 331 MR analyses. Migraine was a risk factor for 3 diseases and a protective factor for 3 diseases, 3 behavioral habits factors, 1 dietary intake factors, and 3 physiologic factors. Migraine had no association with 24 diseases and 5 physiologic factors. Six diseases, 2 behavioral habits factors, and 11 physiologic factors were risk factors for migraine. Three behavioral habits factors, 4 dietary intake factors, and 13 physiologic factors were protective factors of migraine. Fifteen diseases, 6 behavioral habits factors, 1 dietary intake factor, and 8 physiologic factors had no association with migraine. In subtype analysis, we found MOA was a risk factor for 2 diseases, a protective factor for 2 diseases, and 1 behavioral habit factor. MOA had no association with 8 diseases. 1 disease, 1 behavioral habit factor, and 3 physiologic factors were risk factors of MOA. One disease, 2 behavioral habits factors, and 1 physiologic factor were protective factors of MOA. Seven diseases, 2 dietary intake factors, and 2 physiologic factors had no association with MOA. MWA was a risk factor for 1 disease and a protective factor for 1 disease. MWA had no association with 7 diseases. Three diseases and 3 physiologic factors were risk factors of MWA. One behavioral habit factor, 1 dietary intake factor, and 2 physiologic factors were protective factors of MWA. Six diseases, 1 dietary intake factor, and 1 physiologic factor had no association with MWA.

This study also integrated 29 migraine-associated drug targets, including genetic variants linked to smoking behavior, lipid metabolism-related genes, and their expressed proteins. Their expression levels were found to be associated with migraine risk, providing novel insights into the genetic architecture of migraine. However, the pharmacological effects on these targets demonstrate considerable promiscuity and many off-target effects cannot be adequately explored through MR analysis. Further fundamental research and clinical investigations are required to elucidate the potential bioactivities of these significant signals and achieve a more comprehensive understanding.

Some recent studies have revealed that the impact of certain factors on migraine is not direct but rather indirect. For instance, Zhonghua Xiong et al. discovered that atrophy in the subthalamic nucleus subregion plays a critical role in increasing migraine risk, and this effect is partially mediated through alterations in the gut microbiome composition (79). Similarly, Zixiong Shen et al. identified a positive causal relationship between gastroesophageal reflux disease (GERD) and migraine, highlighting the mediating role of depression in increasing migraine risk induced by GERD (80). These findings align to some extent with the results of the present study, yet these researchers delved deeper into the mediating relationships among these factors. This suggests that the migraine-related factors identified in existing research may arise through more complex cascades of mechanisms. Future studies should focus on the interconnections among multiple factors to derive more comprehensive conclusions.

This study has several limitations. First, the analytical framework may lack generalizability across populations with diverse ethnicities or geographical distributions, as the majority of included studies exclusively involved individuals of European ancestry—a constraint inherent to the original data sources. Subsequent data collection and analyses are warranted to validate the universality of findings. Second, the absence of disease severity stratification in case classification precludes assessment of potential associations between migraine progression and clinical severity gradients. Third, there may exist a minimal number of MR investigations utilizing non-overlapping GWAS sources that were not subjected to meta-analysis consolidation. Fourth, our screening protocol did not impose restrictions on the number of SNPs employed in MR analyses, nor did it exclude studies utilizing limited genetic instruments (<10 SNPs). This methodological heterogeneity could introduce estimation biases when integrating such studies with adequately powered investigations during meta-analysis. As MR methodologies continue to evolve, incorporating advanced statistical approaches such as multivariable MR and mediation analysis may help mitigate confounding effects, thereby enhancing the precision of causal inference. Since some included MR studies did not report multiple testing correction, the synthesized results may carry an inflated risk of type I error. This highlights the need for future primary MR studies to adhere to relevant reporting standards and implement comprehensive multiple testing corrections to enhance the reliability of findings.

In addition, some results in this study exhibited substantial heterogeneity. Specifically, high heterogeneity was observed in the analysis investigating the impact of migraine on circulatory system diseases. After removing the outlier (VTE), the I2 value remained high at 71%. We propose that residual heterogeneity may stem from the use of identical genetic instruments/data alongside the combination of outcomes with distinct biological mechanisms. Regarding migraine and non-disease physiological factors, the severe heterogeneity was entirely attributable to the IL-2 data point, as evidenced by the elimination of heterogeneity upon its exclusion. However, the overall effect remained non-significant after exclusion, indicating no causal relationship between migraine and non-disease physiological factors. This heterogeneity likely originated from fundamental differences in exposure characteristics or variations in study design. This finding underscores that combining exposures with divergent biological mechanisms may distort causal inference. Both primary and subgroup analyses of non-disease physiological factors and migraine showed significant heterogeneity (Figure 17). Leave-one-out analysis revealed no substantial changes in results upon sequential removal of individual studies. Potential sources include (1) the inclusion of diverse physiological indicators linked to distinct biological pathways of disease pathogenesis and (2) substantial methodological variations across studies that were difficult to standardize.

Comprehensive analysis indicates that high heterogeneity primarily emerged in the non-disease physiological factors category—a supplementary classification for exposures not covered by ICD-11 disease codes. Although we subcategorized these factors into dietary intake, behavioral habits, and physiological indicators, the latter remains an excessively broad domain encompassing countless physiological mechanisms, rendering the classification inadequate. Current limitations in data richness further impede finer subcategorization. Future studies should therefore conduct systematic and granular stratified analyses of physiological indicators based on underlying biological mechanisms to better elucidate bidirectional causal relationships between physiological markers and migraine.

## 5 Conclusion

This systematic review summarizes the potential causal relationships between migraine and various factors derived from MR studies and also analyzes migraine subtypes. We discuss risk factors and protective factors associated with migraine, both forward and reverse. Moreover, we summarize the genes associated with migraine and their downstream signaling and drug targets, which will provide ideas and directions for future migraine research and bring value to the prevention and treatment of migraine.

## Author’s note

This study conducted the systematic review following the criteria of the PRISMA guidelines and checked against the PRISMA 2020 checklist, which is attached as Supplementary material 3.

## Data Availability

The original contributions presented in the study are included in the article/supplementary material. Further inquiries can be directed to the corresponding author.

## Glossary

MWA: Migraine with aura
MOA: Migraine without aura
NSAID: Non-Steroidal Anti-Inflammatory Drug
MR: Mendelian randomization
IVs: Instrumental variables
IVW: Inverse Variance Weighted
PRISMA: Preferred Reporting Items for Systematic Reviews and Meta-Analyses
PROSPERO: International Prospective Register of Systematic Reviews
CNKI: China National Knowledge Infrastructure
WanFang: Wanfang Data Knowledge Service Platform
VIP: VIP China Science and Technology Journal Database
OR: Odds Ratio
95%CI: 95% Confidence Interval
GWAS: Genome-Wide Association Studies
SNP: Single-nucleotide polymorphism
STROBE-MR: Strengthening the Reporting of Observational Studies in Epidemiology using Mendelian Randomization
ICD-11: International Classification of Diseases, 11th Revision
WHO: World Health Organization
I2: I-squared statistic
AF: Atrial fibrillation
CAD: Coronary artery disease
CeAD: Cervical artery dissection
LAS: Large artery stroke
VTE: Venous thromboembolism
IHGC: International Headache Genetic Consortium
UKB: UK Biobank
FinnGen: Finnish Genome Study
T1D: Type 1 diabetes
T2D: Type 2 diabetes
AD: Alzheimer’s disease
MS: Multiple sclerosis
AR: Allergic rhinitis
RA: Rheumatoid arthritis
SLE: Systemic lupus erythematosus
MD: Meniere’s disease
CKD: Chronic kidney disease
PD: Periodontitis
IGF-1: Insulin-like growth factor 1
IL-2: Interleukin-2
ICV: Intracranial volume
WM: White matter
IDPs: Imaging-derived phenotypes
MDD: Major depressive disorder
SBs: Sedentary behaviors
APTT: Activated partial thromboplastin time
GMV: Gray matter volume
HGF: Hepatocyte growth factor
WMH: White matter hyperintensities
DBP: Diastolic blood pressure
PP: Pulse pressure
SBP: Systolic blood pressure
FVIII: Coagulation factor VIII
vWF: von Willebrand factor
HV: Hippocampal volume
SA: Surface area (cortical)
WMLs: White matter lesions
LDL-C: Low-density lipoprotein cholesterol
APOB: Apolipoprotein B
TC: Total cholesterol
*HMGCR-3*: Hydroxy-3-Methylglutaryl-CoA Reductase
LPL: Lipoprotein lipase
CSD: Cortical spreading depression
CGRP: Calcitonin gene-related peptide
GERD: gastroesophageal reflux disease
