# Supplementary material for: Causal factors for migraine in Mendelian randomization studies: a systematic review and meta-analysis
Source: Front Neurol. 2025 Sep 9;16:1660995. doi: 10.3389/fneur.2025.1660995 (PMC12454037; doi:10.3389/fneur.2025.1660995)
Supplement: Supplementary file 1 [file Table_1.DOCX]

**Supplementary Material 1**

# **Causal Factors for Migraine in Mendelian Randomization Studies: A Systematic Review and Meta‐analysis**

# Frontiers in Neurology

# First author: Xinyao Li

Corresponding authors: Jiaqi Ni, [jiaqini007@163.com](mailto:jiaqini007@163.com%20) & Shu Yang, yangshu1106@scu.edu.cn

**Table of Contents**

**[SM Table 1, Search strategy 2](#_Toc18856)**

**[SM Table 2, Quality assessment scheme 4](#_Toc15684)**

**[SM Table 3, Quality score from individuals studies 9](#_Toc7657)**

## **SM Table 1, Search strategy**

Search strategy

| Database | Search strategy | Result |
| --- | --- | --- |
| Pubmed | ((((((((Migraine*[Title/Abstract]) OR (Hemicrania*[Title/Abstract])) OR (Cephalalgia*[Title/Abstract])) OR (Headache*[Title/Abstract]))) OR (Cephalodynia*[Title/Abstract])) OR (Cranial Pain[Title/Abstract])) OR (Head Pain[Title/Abstract])) AND ((Mendelian Randomization[Title/Abstract]) OR (Mendelian Randomisation[Title/Abstract]) OR (eqtl[Title/Abstract]) OR (pqtl[Title/Abstract]) OR (instrumental variable[Title/Abstract]) OR (genetic instrument[Title/Abstract])) | 96 |
| Embase | #1Migraine*.ab. or Migraine*.ti.  #2 Headache*.ab. or Headache*.ti.  #3 Hemicrania*.ab. or Hemicrania*.ti.  #4 Cephalalgia*.ab. or Cephalalgia*.ti.  #5 Cephalodynia*.ab. or Cephalodynia.ti.  #6 'Cranial Pain'.ab. or 'Cranial Pain'.ti.  #7 'Head Pain'.ab. or 'Head Pain'.ti.  #8 Mendelian Randomization.ab. or Mendelian Randomization.ti.  #9 Mendelian Randomisation.ab. or Mendelian Randomisation.ti.  #10 eqtl.ab. or eqtl.ti.  #11 pqtl.ab. or pqtl.ti.  #12 genetic instrument.ab. or genetic instrument.ti.  #13 instrumental variable.ab. or instrumental variable.ti.  #14 #1 or #2 or #3 or #4 or #5 or #6 or #7  #15 #8 or #9 or #10 or #11 or #12 or #13  #16 #14 and #15 | 115 |
| Cochrane | #1Migraine*.ab. or Migraine*.ti.  #2 Headache*.ab. or Headache*.ti.  #3 Hemicrania*.ab. or Hemicrania*.ti.  #4 Cephalalgia*.ab. or Cephalalgia*.ti.  #5 Cephalodynia*.ab. or Cephalodynia.ti.  #6 'Cranial Pain'.ab. or 'Cranial Pain'.ti.  #7 'Head Pain'.ab. or 'Head Pain'.ti.  #8 Mendelian Randomization.ab. or Mendelian Randomization.ti.  #9 Mendelian Randomisation.ab. or Mendelian Randomisation.ti.  #10 eqtl.ab. or eqtl.ti.  #11 pqtl.ab. or pqtl.ti.  #12 genetic instrument.ab. or genetic instrument.ti.  #13 instrumental variable.ab. or instrumental variable.ti.  #14 #1 or #2 or #3 or #4 or #5 or #6 or #7  #15 #8 or #9 or #10 or #11 or #12 or #13  #16 #14 and #15 | 12 |
| SCI | ((((((((((((((AB=(Migraine*)) OR TI=(Migraine*)) OR AB=(Hemicrania*)) OR TI=(Hemicrania*)) OR AB=(Cephalalgia*)) OR TI=(Cephalalgia*)) OR AB=(Headache*)) OR TI=(Headache*)) OR AB=(Cephalodynia*)) OR TI=(Cephalodynia*)) OR AB=('Cranial Pain')) OR TI=('Cranial Pain')) OR AB=('Head Pain')) OR TI=('Head Pain')) AND (TI=(Mendelian Randomization) OR AB=(Mendelian Randomization) OR TI=(eqtl) OR AB=(eqtl) OR TI=(pqtl) OR AB=(pqtl) OR TI=(Mendelian Randomisationl) OR AB=(Mendelian Randomisation) OR TI=(genetic instrument) OR AB=(genetic instrument) OR TI=(instrumental variable) OR AB=(instrumental variable)) | 157 |
| CNKI | TKA=(偏头痛 or 神经性头痛 or 神经血管性头痛)*(孟德尔随机化) | 4 |
| WanFang | 题名或关键词或摘要：（偏头痛 or 神经性头痛 or 神经血管性头痛）and （孟德尔随机化） | 3 |
| VIP | 题名或关键词或摘要：（偏头痛 or 神经性头痛 or 神经血管性头痛）and （孟德尔随机化） | 2 |
| Total |  | 389 |

## **SM Table 2, Quality assessment scheme**

Quality assessment scheme

| **Item No** | **Section** | **Checklist item** |
| --- | --- | --- |
| 1 | **Title and abstract** | Indicate mendelian randomisation (MR) as the study’s design in the title and/or the abstract if that is a main purpose of the study |
| 2 | Background | Explain the scientific background and rationale for the reported study. What is the exposure? Is a potential causal association between exposure and outcome plausible? Justify why MR is a helpful method to address the study question |
| 3 | Objectives | State specific objectives clearly, including prespecified causal hypotheses (if any). State that MR is a method that, under specific assumptions, intends to estimate causal effects |
| 4 | Study design and data sources | Present key elements of the study design early in the article. Consider including a table listing sources of data for all phases of the study. For each data source contributing to the analysis, describe the following: |
|  | a) | Setting: Describe the study design and the underlying population, if possible. Describe the setting, locations, and relevant dates, including periods of recruitment, exposure, follow-up, and data collection, when available. |
|  | b) | Participants: Give the eligibility criteria, and the sources and methods of selection of participants. Report the sample size, and whether any power or sample size calculations were carried out prior to the main analysis |
|  | c) | Describe measurement, quality control, and selection of genetic variants |
|  | d) | For each exposure, outcome, and other relevant variables, describe methods of assessment and diagnostic criteria for diseases |
|  | e) | Provide details of ethics committee approval and participant informed consent, if relevant |
| 5 | Statistical methods: main analysis | Describe statistical methods and statistics used |
|  | a) | Describe how quantitative variables were handled in the analyses (that is, scale, units, model) |
|  | b) | Describe how genetic variants were handled in the analyses and, if applicable, how their weights were selected |
|  | c) | Describe the MR estimator (eg, two stage least squares, Wald ratio) and related statistics. Detail the included covariates and, in the case of two sample MR, whether the same covariate set was used for adjustment in the two samples |
|  | d) | Explain how missing data were addressed |
|  | e) | If applicable, indicate how multiple testing was addressed |
| 6 | Software and pre-registration |  |
|  | a) | Name statistical software and package(s), including version and settings used |
|  | b) | State whether the study protocol and details were pre-registered (as well as when and where) |
| 7 | Descriptive data |  |
|  | a) | Report the numbers of individuals at each stage of included studies and reasons for exclusion. Consider use of a flow diagram |
|  | b) | Report summary statistics for phenotypic exposure(s), outcome(s), and other relevant variables (eg, means, SDs, proportions) |
|  | c) | If the data sources include meta-analyses of previous studies, provide the assessments of heterogeneity across these studies |
|  | d) | For two sample MR: i. Provide justification of the similarity of the genetic variant-exposure associations between the exposure and outcome samples ii. Provide information on the number of individuals who overlap between the exposure and outcome studies |
| 8 | Main results |  |
|  | a) | Report the associations between genetic variant and exposure, and between genetic variant and outcome, preferably on an interpretable scale |
|  | b) | Report MR estimates of the association between exposure and outcome, and the measures of uncertainty from the MR analysis, on an interpretable scale, such as odds ratio or relative risk per SD difference |
|  | c) | If relevant, consider translating estimates of relative risk into absolute risk for a meaningful time period |
|  | d) | Consider plots to visualise results (eg, forest plot, scatterplot of associations between genetic variants and outcome v between genetic variants and exposure) |
| 9 | Sensitivity analyses and additional analyses |  |
|  | a) | Report any sensitivity analyses to assess the robustness of the main results to violations of the assumptions |
|  | b) | Report results from other sensitivity analyses or additional analyses |
|  | c) | Report any assessment of direction of causal association (eg, bidirectional MR) |
|  | d) | When relevant, report and compare with estimates from non-MR analyses |
|  | e) | Consider additional plots to visualise results (eg, leave-one-out analyses) |
| 10 | Key results | Summarise key results with reference to study objectives |
| 11 | Limitations | Discuss limitations of the study, taking into account the validity of the instrumental variable assumptions, other sources of potential bias, and imprecision. Discuss both direction and magnitude of any potential bias and any efforts to address them |
| 12 | Interpretation |  |
|  | a) | Meaning: Give a cautious overall interpretation of results in the context of their limitations and in comparison with other studies |
|  | b) | Mechanism: Discuss underlying biological mechanisms that could drive a potential causal association between the investigated exposure and the outcome, and whether the gene-environment equivalence assumption is reasonable. Use causal language carefully, clarifying that instrumental variable estimates may provide causal effects only under certain assumptions |
|  | c) | Clinical relevance: Discuss whether the results have clinical or public policy relevance, and to what extent they inform effect sizes of possible interventions |
| 13 | Generalisability | Discuss the generalisability of the study results (a) to other populations, (b) across other exposure periods/timings, and (c) across other levels of exposure |
| 14 | Mendelian Randomisation core assumptions |  |
|  | Assumptions | Explicitly state the three core instrumental variable assumptions for the main analysis (relevance, independence, and exclusion restriction), as well assumptions for any additional or sensitivity analysis |
|  | Assessment of assumptions |  |
|  | a) | Report the assessment of the validity of the assumptions |
|  | b) | Report any additional statistics (eg, assessments of heterogeneity across genetic variants, such as I^2^, Q statistic, or E value) |

## Items are scored as follows: An item that meets the guideline definition is assigned 1 point; a slight deviation from the guideline definition is assigned 0.5 points; and a significant deviation from the guideline definition is assigned 0 points. The maximum score is 14 points. Scores were then converted into percentages, with scores below 80% indicating a high risk of bias, scores between 80% and 90% indicating a medium risk of bias, and scores above 90% indicating a low risk of bias.

## **SM Table 3, Quality score from individuals studies**

Overview of scores from the Quality assessment scheme

| Study (First author, year) | Items | | | | | | | | | | | | | | |
| --- | --- | --- | --- | --- | --- | --- | --- | --- | --- | --- | --- | --- | --- | --- | --- |
|  | 1 | 2 | 3 | 4 | 5 | 6 | 7 | 8 | 9 | 10 | 11 | 12 | 13 | 14 | Total |
| Peter Yin,2017[18] | + | + | . | - | . | + | . | . | . | . | + | + | + | - | 64.3% |
| Johnsen, M. B. 2018[19] | + | + | + | - | - | - | . | . | - | . | . | . | - | - | 39.3% |
| Daghlas, I,2020(1)[20] | + | + | + | + | . | + | . | . | . | . | . | . | + | . | 71.4% |
| Daghlas, I,2020(2)[21] | + | + | + | + | + | + | . | + | + | + | . | + | + | + | 92.9% |
| Daghlas, I,2020(3)[22] | + | + | + | + | . | + | . | + | . | + | . | . | + | . | 78.6% |
| Emmanuel O. Adewuyi,2020[23] | + | + | + | + | + | + | . | . | . | + | + | . | + | + | 85.7% |
| Guo, Y,2020[24] | + | + | + | - | - | - | . | . | - | . | . | + | + | - | 50.0% |
| Chu, S,2021[25] | + | + | + | . | . | + | . | + | . | + | + | . | + | . | 78.6% |
| Guo, Y,2021[26] | + | + | + | + | + | + | . | + | . | + | . | . | + | + | 85.7% |
| Brittany L Mitchell,2022[27] | + | + | + | . | . | + | . | + | - | + | + | + | + | + | 82.1% |
| Chen, H,2022[28] | + | + | + | + | + | + | . | + | - | + | + | . | + | + | 85.7% |
| Daghals, I,2022[29] | . | + | + | + | . | + | . | + | . | . | . | . | + | - | 67.9% |
| Islam, M. R,2022[30] | + | + | . | . | . | - | . | . | . | + | . | . | + | - | 57.1% |
| Keon-Joo Lee,2022[31] | + | + | + | . | . | + | . | . | + | + | + | . | + | . | 78.6% |
| Mei-Jun Shu,2022[32] | + | + | + | . | + | + | . | + | + | + | + | + | + | + | 92.9% |
| Peng-Peng Niu,2022[33] | + | + | + | . | + | + | . | . | . | + | + | + | + | + | 85.7% |
| Reziya Abuduxukuer,2022[34] | + | + | + | + | . | + | . | . | . | + | + | + | + | + | 85.7% |
| Shuai Yuan,2022[35] | + | + | + | + | + | + | + | + | . | + | + | + | + | + | 96.4% |
| Bi, Y,2023[36] | + | + | + | + | + | + | . | + | . | + | + | + | + | . | 89.3% |
| Chong Fu,2023[37] | + | + | + | + | + | + | . | + | + | + | - | + | + | + | 89.3% |
| Fang, T,2023[38] | + | + | + | + | + | + | . | + | + | + | + | . | + | + | 92.9% |
| Guo, X,2023[39] | + | + | + | + | . | + | . | . | + | + | + | + | + | + | 89.3% |
| Huo, J,2023[40] | + | + | + | + | . | + | . | + | + | + | + | + | + | + | 92.9% |
| Horton, M. K. 2023[41] | + | + | + | + | . | + | + | . | . | . | . | + | + | + | 82.1% |
| He, Q,2023[42] | + | + | + | + | + | + | + | + | . | + | + | + | + | + | 96.4% |
| Hua Xue,2023[43] | + | + | + | . | + | + | . | . | . | + | + | + | + | + | 85.7% |
| Hui Zheng,2023[44] | + | + | + | - | . | + | . | + | . | . | + | + | + | + | 78.6% |
| Jin, C,2023[45] | + | + | . | . | + | + | . | + | + | + | + | + | + | + | 89.3% |
| Lei Zhao,2023[46] | + | + | + | . | . | + | + | + | + | + | + | + | + | - | 85.7% |
| Mengmeng Wang,2023[47] | + | + | + | . | + | + | . | . | + | . | + | + | + | + | 85.7% |
| Nike Zoe Welander,2023[48] | + | + | + | . | + | + | . | + | . | . | + | + | + | + | 85.7% |
| Tao Wei,2023[49] | + | + | + | . | + | + | . | + | . | + | + | + | + | + | 89.3% |
| Wenqiang Zhang,2023[50] | + | + | + | . | + | + | . | + | . | . | + | + | + | . | 82.1% |
| Xinhui Liu,2023[51] | + | + | + | . | . | + | + | + | + | . | . | + | + | + | 85.7% |
| Xiaofeng Lv ,2023[52] | + | + | + | + | + | + | . | + | + | + | + | + | + | + | 96.4% |
| Zhen-Ni Zhao,2023[53] | + | + | + | . | + | + | . | + | + | + | + | + | + | . | 89.3% |
| Baranova, A,2024[54] | + | + | + | . | + | + | . | + | . | . | + | . | + | . | 78.6% |
| Chengfeng Xu,2024[55] | + | + | + | + | + | . | + | + | - | + | + | + | + | + | 89.3% |
| Chengcheng Zhang,2024[56] | + | + | + | . | . | + | . | + | . | + | + | + | + | + | 85.7% |
| Danfeng Xu,2024[57] | + | + | + | . | + | + | . | + | + | + | + | + | + | + | 92.9% |
| Geng, C,2024[58] | + | + | + | + | + | + | . | + | + | + | . | . | + | . | 85.7% |
| Guanglu Li,2024[59] | + | + | + | . | + | . | . | + | . | + | + | + | + | + | 85.7% |
| Guoliang Zhu,2024[60] | + | + | + | . | + | + | . | + | + | + | + | + | + | + | 92.9% |
| Hao Lv,2024[61] | + | + | + | + | + | + | . | + | + | + | + | + | + | + | 96.4% |
| Hong, P,2024[62] | + | + | + | + | . | + | + | + | . | + | + | + | + | . | 89.3% |
| Jianxiong Gui,2024[63] | + | + | + | . | . | + | . | + | . | + | . | + | + | . | 78.6% |
| Jareebi, Mohammad A,2024[64] | + | + | + | + | . | + | . | + | . | + | . | + | + | . | 82.1% |
| Jinjin Zhang,2024[65] | + | + | + | . | + | + | . | . | + | + | + | + | + | + | 89.3% |
| Kang Qu,2024(1)[66] | + | + | + | . | + | + | . | + | . | + | + | + | + | + | 89.3% |
| Kang Qu,2024(2)[67] | + | + | + | . | + | + | . | + | . | + | + | + | + | + | 89.3% |
| Kangjia Zhang,2024[68] | + | + | + | . | + | + | . | + | + | + | + | + | + | + | 92.9% |
| Lei Zhao,2024[69] | + | + | + | . | + | + | + | + | + | + | + | + | + | + | 96.4% |
| Meixuan Ren,2024[70] | + | + | + | . | + | + | + | + | + | + | + | + | + | + | 96.4% |
| Peihong Li,2024[71] | + | + | + | . | + | + | . | . | . | + | + | + | + | + | 85.7% |
| Peng-Peng Niu,2024[72] | + | + | + | . | + | + | . | + | . | + | + | + | + | + | 89.3% |
| Xiangyue Meng,2024[73] | + | + | + | . | + | + | + | + | + | + | + | + | + | + | 96.4% |
| Xu-Peng Wu,2024[74] | + | + | + | . | + | + | + | + | . | + | + | + | + | + | 92.9% |
| Ya Li ,2024[75] | + | + | + | . | + | + | . | . | . | + | + | . | + | + | 82.1% |
| Yang Li,2024[76] | + | + | + | + | + | + | . | + | + | + | + | + | . | + | 92.9% |
| Yang Wang,2024[77] | + | + | + | . | + | + | + | + | . | + | + | + | + | + | 92.9% |

Under item 1~14, the assessment criteria are derived from the study by Ibrahim et al [16], and are as follows: title and abstract, background, objectives, study design and data sources, main statistical methods of analysis, software and pre-registration, descriptive data, main results, sensitivity and additional analyses, main results, limitations, interpretation, generalizability, and Mendelian randomization core assumptions.

In the risk of bias table, "+" indicates that the item meets the guideline definition and is assigned 1 point; "." indicates a slight deviation from the guideline definition and is assigned 0.5 points; "-" indicates a significant deviation from the guideline definition and is assigned 0 points.
